# Smoking and infertility: multivariable regression and Mendelian randomization analyses in the Norwegian Mother, Father and Child Cohort Study

**DOI:** 10.1016/j.fertnstert.2022.04.001

**Published:** 2022-05-10

**Authors:** Álvaro Hernáez, Robyn E. Wootton, Christian M. Page, Karoline H. Skåra, Abigail Fraser, Tormod Rogne, Per Magnus, Pål R. Njølstad, Ole A. Andreassen, Stephen Burgess, Deborah A. Lawlor, Maria Christine Magnus

**Affiliations:** a Centre for Fertility and Health, Norwegian Institute of Public Health, Oslo, Norway; b Consorcio CIBER, M.P. Fisiopatología de la Obesidad y Nutrición, Instituto de Salud Carlos III, Madrid, Spain; c Blanquerna School of Health Sciences, Universitat Ramon Llull, Barcelona, Spain; d MRC Integrative Epidemiology Unit, University of Bristol, Bristol, United Kingdom; e Nic Waals Institute, Lovisenberg Diaconal Hospital, Oslo, Norway; f Department of Mathematics, University of Oslo, Oslo, Norway; g Population Health Sciences, Bristol Medical School, University of Bristol, Bristol, United Kingdom; h The National Institute for Health Research Bristol Biomedical Research Centre, Bristol, United Kingdom; i Department of Chronic Disease Epidemiology, Yale University School of Public Health, New Haven, Connecticut; j Department of Circulation and Medical Imaging, Gemini Center for Sepsis Research, NTNU Norwegian University of Science and Technology, Trondheim, Norway; k Department of Clinical Science, Center for Diabetes Research, University of Bergen, Bergen, Norway; l Children and Adolescent Clinic, Haukeland University Hospital, Bergen, Norway; m Division of Mental Health and Addiction, Norwegian Centre for Mental Disorders Research, NORMENT, Oslo University Hospital, Oslo, Norway; n Institute of Clinical Medicine, University of Oslo, Oslo, Norway; o Medical Research Council, Biostatistics Unit, University of Cambridge, Cambridge, United Kingdom; p Cardiovascular Epidemiology Unit, Department of Public Health and Primary Care, University of Cambridge, Cambridge, United Kingdom

**Keywords:** Smoking, infertility, Mendelian randomization

## Abstract

**Objective:**

To investigate the association between smoking and infertility.

**Design:**

Prospective study.

**Setting:**

Nationwide cohort.

**Patients:**

28,606 women and 27,096 men with questionnaire and genotype information from the Norwegian Mother, Father, and Child Cohort Study.

**Intervention:**

Self-reported information on smoking (having ever smoked [both sexes], age at initiation [women only], cessation [women only], and cigarettes/week in current smokers [both sexes]) was gathered. Genetically predetermined levels or likelihood of presenting these traits were estimated for Mendelian randomization.

**Main outcome measure:**

Infertility (time-to-pregnancy ≥ 12 months).

**Results:**

Having ever smoked was unrelated to infertility in women or men. Higher smoking intensity in women was associated with greater infertility odds (+1 standard deviation [SD, 48 cigarettes/week]: odds ratio [OR]_crude_, 1.19; 95% confidence interval [CI] 1.11-1.28; OR_adjusted_ 1.12; 95% CI, 1.03–1.21), also after adjusting for the partner’s tobacco use. Later smoking initiation (+1 SD [3.2 years]: OR_crude_, 0.94; 95% CI, 0.88–0.99; OR_adjusted_ 0.89; 95% CI, 0.84–0.95) and smoking cessation (vs. not quitting: OR_crud_e, 0.83; 95% CI, 0.75–0.91; OR_adjusted_, 0.83; 95% CI, 0.75–0.93) were linked to decreased infertility in women. Nevertheless, Mendelian randomization results were not directionally consistent for smoking intensity and cessation and were estimated imprecisely in the 2-sample approach. In men, greater smoking intensity was not robustly associated with infertility in multivariable regression and Mendelian randomization.

**Conclusions:**

We did not find robust evidence of an effect of smoking on infertility. This may be due to a true lack of effect, weak genetic instruments, or other kinds of confounding. (Fertil Steril® 2022;118:180-90. ©2022 by American Society for Reproductive Medicine.)

**El resumen está disponible en Español al final del artículo.**

## Abstract (fr)

**Objetivo:**

Investigar la asociacion entre el tabaquismo y la infertilidad.

**Diseño:**

Estudio prospectivo.

**Ámbito:**

Cohorte nacional.

**Pacientes:**

28 606 mujeres y 27 096 hombres con información del cuestionario y el genotipo del estudio de cohorte noruego de madres, padres e hijos.

**Intervención:**

Se recopiló información autoinformada sobre el tabaquismo (haber fumado alguna vez [ambos sexos], edad de inicio [solo mujeres], abandono [solo mujeres] y cigarrillos/semana en fumadores actuales [ambos sexos]). Los niveles predeterminados genéticamente o la probabilidad de presentar estos rasgos se estimaron para la aleatorización mendeliana.

**Medida de resultado principal:**

Infertilidad (tiempo hasta el embarazo ≥ 12 meses).

**Resultados:**

Haber fumado alguna vez no estuvo relacionado con la infertilidad en mujeres u hombres. Una mayor intensidad de tabaquismo en las mujeres se asoció con mayores probabilidades de infertilidad (+ 1 desviación estóndar [DE, 48 cigarrillos/semana]: razón de probabilidades [OR]_crudo_, 1.19; intervalo de confianza [IC] del 95%: 1.11–1.28; OR_ajustado_ 1.12; IC del 95%, 1.03–1.21), tambión despues de ajustar el consumo de tabaco de la pareja. Inicio tardío del tabaquismo (+ 1 DE [3.2 años]: OR_crudo_, 0.94; IC del 95%, 0.880.99; OR_aj_·_ustado_ 0.89; IC 95%, 0.84–0.95) y el abandono del habito de fumar (frente a no dejar de fumar: OR_crudo_, 0.83; IC 95%, 0.75–0.91; OR_ajustado_, 0.83; IC 95%, 0.75–0.93) se relacionaron con una disminución de la infertilidad en las mujeres. Sin embargo, los resultados de la aleatorización mendeliana no fueron direccionalmente consistentes para la intensidad y el abandono del hábito de fumar y se estimaron de manera imprecisa en el enfoque de 2 muestras. En los hombres, una mayor intensidad de tabaquismo no se asoció sólidamente con la infertilidad en la regresión multivariable y la aleatorización mendeliana.

**Conclusiones:**

No encontramos pruebas sólidas de un efecto del tabaquismo sobre la infertilidad. Esto puede deberse a una verdadera falta de efecto, instrumentos genéticos dóbiles u otros tipos de confusión.

**S**moking is a well-known source of thousands of prooxidative and proinflammatory compounds (1), capable of damaging reproductive tissues, which in turn may compromise fecundity (2–4). Observational studies in women have reported that active smoking was linked to 14% greater odds of infertility (trying to establish a pregnancy after ≥ 12 months of regular, unprotected sexual intercourse) (5) and smoking intensity was dose-dependently associated with greater infertility risk (6). Tobacco use also has been related to surrogate indicators of decreased fertility, such as accelerated follicular depletion and earlier menopause (7, 8). Although smoking has been linked to oligozoospermia and poor sperm quality (9–11), 2 prospective studies reported no association with infertility in men (12, 13). Considering this evidence, the Practice Committee of the American Society for Reproductive Medicine has suggested a causal effect of tobacco use on infertility (14). However, existing evidence is subject to numerous methodologic limitations. The primary concerns raised were that the relationship between smoking and decreased fertility has not been shown to be sufficiently strong, residual confounding cannot be ruled out, and most studies were retrospective and, thus, unable to reveal any potential exposure-to-effect sequence (14).

The use of complementary methodologic approaches with different strengths and sources of bias could help clarify whether there is a causal relationship between smoking and infertility (15). Mendelian randomization (MR) is based on the use of genetic variants that are linked to an exposure (e.g., having ever smoked) to assess the unconfounded effect of this exposure on a certain outcome (e.g., infertility) (16). It can be performed using data from a single sample (1-sample MR: exposure, outcome, and genetic variants are measured in the same population) or from 2 samples (2-sample MR: the association between genetic variants and the exposure are assessed in 1 population and the association between genetic variants and the outcome in a second population) (17). In this study, we compare results from multivariable logistic regression, 1-sample MR, and 2-sample MR, affected by different and unrelated sources of bias (15) (Table 1). Similar results in all of them would allow for more robust conclusions (15).

We hypothesize that there may be a possible causal relationship between smoking and infertility. Our aim was to investigate the association between tobacco use and infertility in women and men by multivariable logistic regression and 1- and 2-sample MR.

## Material and Methods

### Study Participants

We used data from the Mother, Father, and Child Cohort Study (MoBa) (18, 19). The MoBa Study is a population-based pregnancy cohort study conducted by the Norwegian Institute of Public Health. Pregnant women and their partners across Norway were recruited between 1999 and 2008 at the time of the routine ultrasound screening (approximately 18th gestational week). The cohort now includes 114,500 children, 95,200 mothers, and 75,200 fathers. Our work is based on version #12 of the quality-assured data.

We defined a subsample of parents involved in singleton pregnancies with available genotype data and prepregnancy information on tobacco use. Our genotype data came from blood samples gathered from parents during pregnancy (20) and followed the pipeline described by Helgeland et al. (21) (genotype calling, imputation, and quality control). Our work is described according to the Strengthening the Reporting of Observational studies in Epidemiology guidelines for reporting MR and cohort studies (22, 23).

### Tobacco Use

Parents responded to questions related to their smoking habits at recruitment. First, they reported if they had ever smoked. After an affirmative answer, participants reported their age when they started smoking, if they were smokers when they got pregnant, if they had quit smoking (and the date when they quit), and the number of cigarettes/week they currently smoked or used to smoke. Using these data, we defined 3 exposure variables: ever smoker (yes/no, available for the whole population), age at initiation (continuous, available in current/former female smokers), and cessation (yes/no, available in current/former female smokers). In addition, we computed the average cigarettes smoked per week at the moment or during the 2 years before getting pregnant (continuous, available in current smokers, and in participants who smoked at this time).

### Selection of Genetic Variants and Generation of Instrumental Variables

Genetic instruments were extracted from the most recent genome-wide association study (GWAS) by Liu et al. (24) on smoking-related traits. It included > 1.2 million individuals (none of them participated in MoBa) and reported 378 independent single nucleotide polymorphisms (SNPs) associated with smoking initiation, 55 linked with smoking heaviness (cigarettes/week), 10 related to the age at smoking initiation, and 24 associated with smoking cessation (24). Independent SNPs were defined according to linkage disequilibrium blocks across the genome, presented a minor allele frequency ≥ 0.1% and were associated with their respective phenotypes according to the standard genome-wide significance threshold (*P*<5 × 10^−8^)(24). 355 (94%) of the SNPs in the study of Liu et al. (24) GWAS were available for smoking initiation in the MoBa genotype database, 50 (91%) for smoking heaviness, 10 (100%) for age at smoking initiation, and 23 (96%) for smoking cessation, respectively.

For the 1-sample MR analyses, we generated a weighted genetic risk score (GRS) by multiplying the number of risk alleles by the effect estimate of each variant and dividing this value by the total number of SNPs (25). Single nucleotide polymorphisms were used individually as the genetic instruments in the 2-sample MR. Information on which SNPs were considered in 1-sample and 2-sample MR analyses is available in Supplemental Table 1 (available online).

### Infertility

Women were asked at recruitment if the pregnancy was planned and to provide information on how many months they had been trying to get pregnant (19). The answer options were “<1 month,” “1–2 months,” and “≥3 months.” If the mother gave this last answer, she was asked to specify further how many months. We defined infertility as trying to establish a pregnancy after ≥ 12 months of regular, unprotected sexual intercourse or having used assisted reproductive technologies (ARTs). Participants being offered ART treatments in the public Norwegian system should have tried to get pregnant for ≥ 12 months (26). Those reporting trying for <12 months (fertile) were included in the reference group. Participants involved in unplanned pregnancies were excluded.

### Other Variables

Information on the age of the participants (continuous), educational level (years of education equivalent to the US system (27), continuous), prepregnancy body mass index (BMI, continuous), and previous number of deliveries (0, 1, 2, or ≥ 3) was gathered in the baseline visit as potential confounders. Further lifestyle- and occupation-related covariates also were considered (fiber intake [a proxy for diet quality], physical activity levels, caffeine intake, alcohol use, and exposure to occupational toxicants [yes/no]), and the information available on these lifestyle characteristics is described in Supplemental Methods (available online).

### Sample Size

The number of participants and infertility cases in our study gave us adequate power (2-sample *α* risk = 0.05, power ≥80%) to detect odds ratios (ORs) of 1.11 for smoking initiation in women, 1.11 for smoking initiation in men, 1.10 for smoking intensity in women, 1.11 for smoking intensity in men, 0.86 for smoking cessation in women, and 0.92 for age for smoking initiation in women (Supplemental Tables 2 and 3, available online).

### Statistical Analyses

Normally-distributed continuous variables were described by means and SD, nonnormally distributed continuous variables by medians and 25th–75th percentiles, and categorical variables by proportions. Differences in baseline characteristics between infertile and fertile parents, and between participants with and without genotype information in MoBa, were investigated by Student’s *t* tests in normally-distributed continuous variables, Mann-Whitney *U* tests in nonnormally distributed continuous variables, and χ^2^ tests in categorical variables.

#### Multivariable regression analyses

We assessed the association between smoking-related traits and infertility in women and men by multivariable logistic regressions. For binary exposures (smoking initiation, smoking cessation), we investigated the differences in the odds of infertility in those exposed compared to those who were not. For continuous variables, we assessed the relationship between an increase in 1 SD in the number of cigarettes smoked per week (in current smokers) and the age at smoking initiation (in ever smokers) with odds of infertility. We also assessed whether a model using smoothed cubic splines (*K*+4 degrees of freedom) fitted the data better than a linear function using a likelihood ratio test. All models were crude and adjusted for predefined infertility risk factors: age, years of education, BMI, and number of previous pregnancies. We did a complete case analysis without multiple imputation. We further adjusted them for the partner’s trait in those traits in which there was information from both parents (smoking initiation and intensity) to minimize bias due to assortative mating, and lifestyle (diet quality, physical activity, caffeine, and alcohol use) and occupational features in additional sensitivity analyses. Clustered standard errors were computed to account for nonindependence between pregnancies in participants involved in >1 pregnancy in MoBa (1 pregnancy: 18,137 women and 18,243 men; ≥2 pregnancies: 5,113 women and 4,337 men).

As sensitivity analyses, we studied the association between smoking-related traits and infertility according to whether they used ART or not.

#### One-sample Mendelian randomization

We used logistic regression to estimate the genetically predicted likelihood of smoking initiation (both sexes, all participants) and smoking cessation (women, ever smokers), and linear regression to calculate the genetically predicted values of the number of cigarettes smoked per week (both sexes, current smokers) and the age at smoking initiation (women, ever smokers), using their respective GRSs as predictors. Next, we assessed the linear relationship between an increase in 1 SD in the genetically predicted traits and infertility using logistic regression.

#### Two-sample Mendelian randomization

We first performed 2 GWASs (1 for women and 1 for men) to obtain summary associations of each SNP with infertility in the MoBa cohort. Full details are available in the Supplemental Methods. In the GWAS summary data, we looked for the SNPs related to each of the smoking traits and extracted the information related to their association with infertility. We harmonized both datasets and excluded palindromic SNPs with minor allele frequencies close to 0.5 (17). After the harmonization, 301 (80%) of the SNPs in the study of Liu et al. (24) GWAS were considered for smoking initiation, 43 (78%) for smoking heaviness, 7 (70%) for age at smoking initiation, and 16 (67%) for smoking cessation (Supplemental Table 1). We used inverse variance weighted regression as the main 2-sample MR analysis (17).

#### Verification of MR assumptions

The key assumptions of MR are: the genetic instrument is related robustly to the exposure, the genetic instrument is associated only with the outcome through the exposure of interest, and there is no confounding of the genetic instrument-outcome associations (17).

Regarding the first assumption, we checked the strength of the association between the genetic instruments and their phenotypes. For binary exposures, we used logistic regression, area under the receiver operating characteristic (ROC) curve, and pseudo-*R^2^* by the Nagelkerke method, and for continuous exposures we used linear regressions, *F* statistics and *R^2^.* Since weak instruments deviate MR causal estimates toward the null in 2-sample MR, concordance between 1-sample and 2-sample MR reduces the risk of weak instrument bias (28). We also performed the Robust Adjusted Profile Score 2-sample MR method, unbiased by weak instruments (28, 29). The second assumption may be violated when the genetic instruments influence other risk factors for the outcome independently of the exposure of interest (horizontal pleiotropy) (30). To evaluate horizontal pleiotropy in 1-sample MR, we checked the association of GRSs with known risk factors for infertility. We studied the relationship between 1 SD increase in the GRS and the risk factors (age, years of education, BMI, number of previous deliveries) (31, 32) using linear regressions. If any of the GRSs was associated with a risk factor, we considered that a potential pleiotropic effect was present for all the smoking-related traits. We then performed multivariable MR analyses if GWAS data for the potential pleiotropic variable were available (33). There was evidence of ≥ 1 of the smoking trait GRS associating with education and BMI and we were able to undertake multivariable MR for both. For education, we used the GWAS by Lee et al. (34) (n = 1,271 independent SNPs, approximately 1.1 million participants; 1,159 of the SNPs were available in MoBa). For BMI we used the GWAS by Yengo et al. (35) (n = 941 independent SNPs, approximately 700,000 participants; 896 of the SNPs were available in MoBa). We generated GRS for education and BMI using the same method as used for the smoking traits and then included the GRS for education and BMI in the 1-sample MR regression models (33). The genetic smoking instruments also were associated with age, and we performed stratified analyses according to age (below vs. over the median). In addition, we estimated the association between the GRSs for smoking traits and infertility in nonexposed participants (the GRSs for age at smoking initiation or smoking cessation in never smokers, and the GRS for current smoking intensity in never + former smokers). As we would expect no association in nonexposed participants, any evidence of one would indicate the presence of bias.

In 2-sample MR we explored unbalanced horizontal pleiotropy comparing the main estimates to those obtained from MR-Egger, the weighted median and weighted mode methods (36). The inverse variance weighted method assumes no unbalanced horizontal pleiotropy as it forces the regression line through SNP-exposure and SNP-outcome coordinates to go through 0. Mendelian randomization-Egger does not make this assumption and it does not force the line through 0. A nonzero intercept is an indication of unbalanced horizontal pleiotropy, and the slope subsequently is corrected. The weighted median and weighted mode analyses are valid if <50% of the weight comes from SNPs that are not related to other risk factors for the outcome. Concordance in the estimates across the different approaches reduces the concern regarding unbalanced pleiotropy (36). We also checked for influential outliers in the variant-specific causal estimates (indicative of horizontal pleiotropy) in scatterplots. Finally, we evaluated between SNP heterogeneity, using Cochran’s *Q* and the Rücker’s *Q’* with the inverse variance weighted and Egger regression methods, respectively. Heterogeneity indicates a possible violation of the MR assumptions, of which pleiotropy probably is a major cause (36).

To reduce the potential for confounding of the genetic instrument-outcome association due to population stratification (third assumption), we adjusted for the first 10 ancestry-informative principal components in the 1-sample MR (37).

#### Software

Analyses were performed in R Software version 4.0.3 (packages: *compareGroups, estimatr, ggplot2, miceadds,* and *TwoSampleMR*) and the GWASs to determine which SNPs were associated with infertility in the MoBa cohort in Plink v1.9 and GWAMA (38, 39). Code for data management and statistical analysis has been made available in https://github.com/alvarohernaez/MR_smoking_subfertility_MoBa/.

## Results

### Description of the Study Population and Genetic Instruments

**Figure 1:**
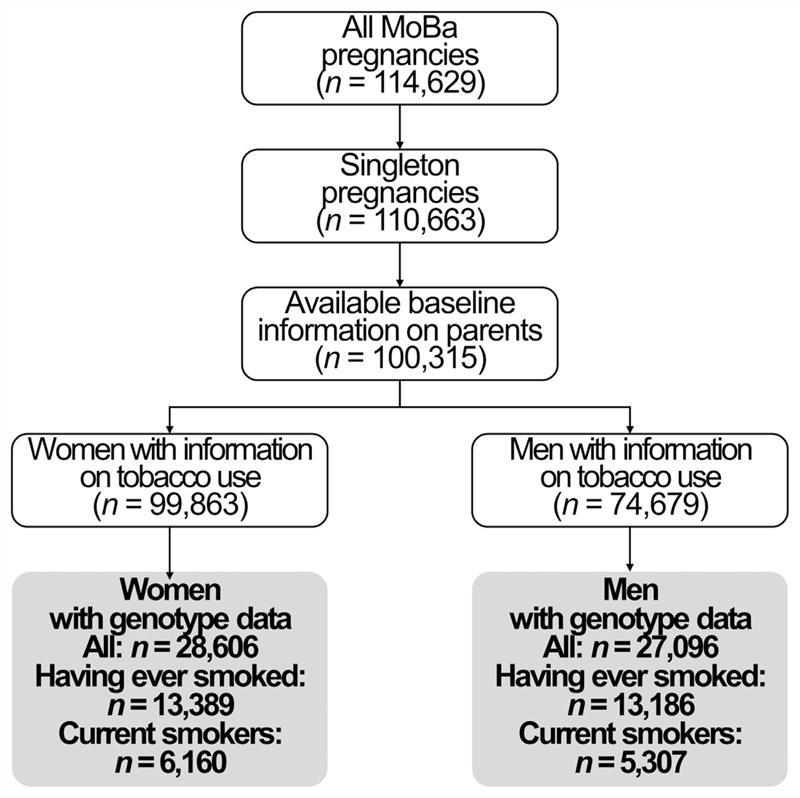
Study flow chart. MoBa = the Mother, Father, and Child Cohort Study.

Our study population consisted of 28,606 women and 27,096 men with genotype information (Fig. 1). A total of 10% of the couples were infertile (of these, 21% underwent ART treatment). Women and men who were infertile were older, had a lower educational attainment, had a higher BMI, and were more likely to be pregnant for the first time. There was a higher proportion of ever smokers in infertile couples and, among tobacco users, smoking intensity was higher in infertile individuals (Table 2). The Mother, Father, and Child Cohort Study participants without genotype data were not meaningfully different from those with genetic data in age, years of education, BMI, or number of previous pregnancies. However, participants presented a different proportion of ever smokers (women were less likely and men were more prone to have ever smoked) and showed a lower smoking intensity among current smokers (Supplemental Table 4, available online).

Genetic risk scores for smoking initiation were linked identically to having ever smoked in both sexes (1 point increase in the GRS, women: OR, 1.02; 95% confidence intervals [CI], 1.018–1.022, 0.57 area under the ROC curve; men: OR, 1.02; 95% CI, 1.017–1.022, 0.56 area under the ROC curve). Genetic risk scores for smoking intensity were related to the number of cigarettes smoked per week in a similar way in both sexes (1 point increase in the GRS, women: +0.80 cigarettes/week; 95% CI, 0.62–0.98, *F* statistic 76; men: +0.87 cigarettes/week; 95% CI, 0.66–1.09, *F* statistic 64). The GRS for smoking cessation was associated with greater odds of quitting in women (1 point increase in the GRS, women: OR, 1.03; 95% CI, 1.019–1.047, 0.52 area under the ROC curve). However, the association between age at smoking initiation and its GRS was unclear (1 point increase in the GRS: +0.023 years; 95% CI, −0.014–0.059, *F* statistic 1).

### Comparison of Main Multivariable Regression, 1-Sample, and 2-Sample MR Results

**Figure 2:**
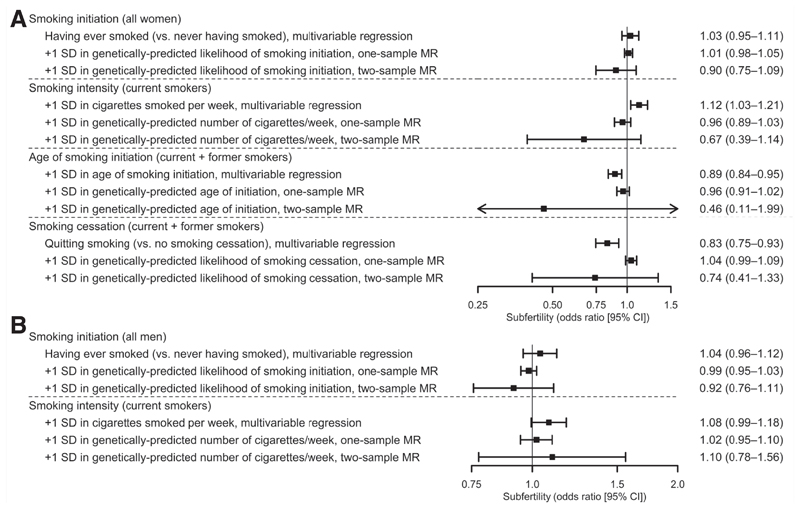
Association between smoking-related traits and infertility in women (**A**) and men (**B**). Results are presented as odds ratios and 95% confidence intervals. CI = confidence interval; MR = Mendelian randomization.

As shown in Figure 2A, smoking initiation was unrelated to infertility in women in all analytic approaches. Regarding smoking intensity, it was linearly linked to greater odds of infertility in multivariable regression (1 SD increase in the number of cigarettes smoked per week [+48 cigarettes/ week]: OR, 1.12; 95% CI, 1.03–1.21; *P*_non-linearity_= .970). The association was consistent for both infertility subtypes (Supplemental Table 5, available online). However, adjusting for the partner’s smoking heaviness slightly attenuated it (OR, 1.10; 95% CI, 1.01–1.19), as did the adjustment for lifestyle and occupational features (OR, 1.08; 95% CI, 0.99–1.19; Supplemental Table 6, available online), close to the null results were observed in 1-sample MR. (OR, 0.96; 95% CI, 0.89–1.03) and a directionally nonconcordant, imprecise estimate was noted in 2-sample MR (OR, 0.67; 95% CI, 0.39–1.14). For age at smoking initiation, we observed a linear association between later smoking initiation and lower odds of infertility in multivariable analyses (1 SD increase in the age at smoking initiation in ever smokers [+3.2 years]: OR, 0.89; 95% CI, 0.84–0.95; *P*_non-linearity_= .933), in both infertility subtypes (Supplemental Table 5) and after adjusting for lifestyle and occupational traits (Supplemental Table 6), close to the null results in 1-sample MR (OR, 0.96; 95% CI, 0.91–1.02), and a greater, imprecise association in 2-sample MR (OR, 0.46; 95% CI, 0.11–1.99). Finally, for cessation among those who had ever smoked, there were similar inverse associations in multivariable regression (OR, 0.83; 95% CI, 0.75–0.93), also after adjusting for lifestyle and occupational features, and 2-sample MR (OR, 0.74; 95% CI, 0.41–1.33), the latter with wide CIs, whereas the result in 1-sample MR was nondirectionally concordant and close to the null (OR, 1.04; 95% CI, 0.99–1.09). Women who quit smoking were less prone to be infertile but not use ART (OR, 0.71; 95% CI, 0.63–0.80) but more likely to use ART (OR, 1.82; 95% CI, 1.40–2.36; Supplemental Table 5).

Smoking initiation also was unrelated to infertility in men (Fig. 2B). However, a linear association between higher smoking intensity and greater odds of infertility was suggested in multivariable analyses (1 SD increase in the number of cigarettes smoked per week [+54 cigarettes/week]: OR, 1.08; 95% CI, 0.99–1.18; *P*_non-__linearity_= .123), attenuated when adjusting for the partner’s smoking intensity (OR, 1.05; 95% CI, 0.96–1.15; Supplemental Table 7, available online). A similar but imprecise association was observed in 2-sample MR (OR, 1.10; 95% CI, 0.78–1.56), while the relationship was close to the null in 1-sample MR (OR, 1.02; 95% CI, 0.95–1.10).

### Verification of MR Assumptions

We observed associations between some smoking-related GRSs and age (in women), education years (in both sexes) and BMI (in both sexes) (Supplemental Table 8, available online). Results of multivariable 1-sample MR accounting for education and BMI and age-stratified analyses were consistent with the main analyses (Supplemental Table 9, available online). No associations between the GRSs for smoking traits and infertility were found in nonsmokers (Supplemental Table 10, available online).

Regarding 2-sample MR additional methods, between SNP heterogeneity, potentially linked to horizontal pleiotropy, was observed for the genetic instrument for smoking intensity in women, as well as highly imprecise MR estimates (Supplemental Table 11, available online, Supplemental Fig. 1, available online).

## Discussion

We found close to the null associations between smoking initiation and infertility in women and men. Smoking heaviness was linked to greater odds of infertility in both sexes in multivariable analyses, although these relationships were attenuated after adjusting for lifestyle, occupational features, and the partner’s smoking intensity, and were nonconcordant with 1-sample and 2-sample MR analyses. Later initiation of smoking and cessation were related to higher odds of infertility in women in multivariable regression, but these associations also were inconsistent with the MR analyses. Overall, a strong effect of smoking on infertility was not observed.

A link between smoking and infertility has been reported in human studies since the 1980s, particularly in women (5, 6). Nevertheless, the American Society for Reproductive Medicine warned in 2018 of some methodologic flaws in the available body of evidence including the risk of residual confounding, the small magnitude of the association, and the retrospective nature of most studies (16). We addressed these limitations here by assessing the association between several smoking-related traits and infertility in a large prospective study comparing multivariable regression results to those from 1-sample and 2-sample MR, undertaking several sensitivity analyses to explore possible sources of bias. Smoking was related to increased odds of infertility in women in our multivariable regression analyses, similarly to previous studies (6). These relationships were of modest magnitude, presented a curtailed risk of bias by assortative mating (40), and showed greater power than MR analyses. Confidence intervals for estimates for smoking intensity and age at initiation (which are directly comparable) overlapped, suggesting the sets of results were consistent with each other. Regarding men, we did not find any robust relationship between smoking and infertility across methods, in agreement with previous prospective studies (12, 13). However, MR results generally were close to the null or not directionally concordant with observational estimates. It is possible that residual confounding due to poorer health status or other unmeasured parameters could explain the decreased fertility among female smokers, as the association between a greater smoking intensity in women who smoked and infertility risk was modestly attenuated after adjusting for other lifestyle and occupational traits and the partner’s tobacco use. The genetic instrument for age at smoking initiation in women was weak, and the lack of phenotype information on smoking cessation and age at smoking initiation in men did not allow us to perform MR analyses. In addition, results were particularly imprecise in the 2-sample MR. Taken together, our results did not provide robust evidence for effects of smoking on infertility.

Our work has some limitations. First, infertility is a couple-dependent parameter that was reported by mothers in the cohort, and we could not determine the cause (female and/or male causes). Second, MoBa is a pregnancy cohort, only including couples who were able to get pregnant. Further studies considering sterile couples are needed. Infertility is a less severe manifestation of sterility and, therefore, an association between smoking-related traits and an absolute incapacity to become pregnant may be possible. Third, our results from the MR analyses may have been affected by selection bias as there were some differences in smoking habits between participants with and without genetic data (included women were less likely to have ever smoked, and included women and men smoked less intensely) and smokers were already underrepresented in the MoBa cohort relative to the whole Norwegian population (41). This may decrease our external validity and our capacity to observe associations (our participants were less exposed than the average population). Nevertheless, associations between smoking and pregnancy outcomes proved unaffected by selection bias in prior studies when the MoBa cohort and the general Norwegian population were compared (41). Fourth, the instrument for age at smoking initiation in women was weak. Any relationship between this trait and infertility seen in 1-sample MR but not in 2-sample MR would be suspicious of being explained by weak instrument bias (Table 1). However, no robust association of this trait with infertility was found in MR analyses. Fifth, there was no phenotype information on age at smoking initiation and smoking cessation in men in MoBa. Therefore, we were not able to check the association between these traits and infertility in males, validate the robustness of their genetic instruments, or perform full MR analyses. Sixth, we observed nondirectionally concordant associations between smoking cessation and infertility without and with the use of ART. This apparent contradiction may be due to reverse causation as women intending to undergo ART may receive a strict recommendation of quitting smoking before starting it (26). Finally, our study population (adult women and men of a northern European ancestry who could get pregnant) restricts the generalizability. However, our work also presents several strengths. To our knowledge, this is the first prospective study assessing the relationship between smoking and infertility using 3 complementary approaches affected by different sources of bias that have been investigated thoroughly and acknowledged. In addition, our study includes a relatively homogeneous population. This aspect minimizes the risk of confounding due to population stratification in MR, together with the adjustment for the first 10 principal components (37).

## Conclusion

In conclusion, we did not find robust evidence of an effect of smoking on infertility. This may be due to a true lack of effect, weak genetic instruments, or other kinds of confounding. However, the comparison of different analytic approaches with complementary sources of bias has highlighted relevant limitations across all methods, and in particular highlights the needs for larger studies with information on infertility.

## Supplementary Material

Supplementary file

## Figures and Tables

**Table 1:** Comparison among multivariable logistic regression, 1- and 2-sample Mendelian randomization regarding sources of bias in the association between smoking and infertility.

	Residual confounding (the effect of smoking on infertility could be due to behavioral or socioeconomic conditions intimately related to smoking, such as poor diet or poorer health status)	Weak genetic instruments (the available genetic instruments for smoking traits explain a small proportion of the variability of the exposure)	Horizontal pleiotropy (the genetic instruments for smoking traits influence the risk of infertility via mechanisms other than smoking)	Confounding of the genetic instrument-outcome association (by population stratification)
Multivariable regression	High risk	Unaffected	Unaffected	Unaffected
1-sample Mendelian randomization	Low risk	Overestimated associations. Consistence with 2-sample Mendelian randomization estimates minimizes the risk of this bias	Possibility to explore the association between genetic instruments and other infertility risk factors. Multivariable Mendelian randomization could then be used to correct for such bias	Adjustment for ancestry-informative principal components minimizes the risk of this bias
2-sample Mendelian randomization	Low risk	Bias toward the null association. The Robust Adjusted Profile Score approach is immune to this bias	Consistence among Mendelian randomization methods, lack of between single nucleotide polymorphism heterogeneity, and lack of outliers in Mendelian randomization scatterplots minimizes the risk of this bias	High risk

**Table 2:** Baseline characteristics and smoking-related properties of study population.

		Women				Men		
	All (n = 28,606)	Infertility reported (n = 3,439)	No infertility reported (n = 25,167)	*P* value	All (n = 27,096)	Infertility reported (n = 3,275)	No infertility reported (n = 23,821)	*P* value
Age (years), mean ± SD	30.3 ± 4.15	31.5 ± 4.36	30.2 ± 4.09	< .001	32.7 ± 4.90	34.1 ± 5.36	32.6 ± 4.81	< .001
Education years, mean ± SD	17.5 ± 3.11	17.0 ± 3.33	17.6 ± 3.08	< .001	16.6 ± 3.50	16.2 ± 3.54	16.6 ± 3.49	< .001
Body mass index (kg/ m^2^), median (25th–75th percentiles)	23.1 (21.2–25.9)	23.7 (21.5–27.2)	23.1 (21.1–25.7)	< .001	25.5 (23.7–27.7)	25.8(24.0–28.1)	25.4(23.7–27.7)	< .001
Previous pregnancies n (%):				< .001				< .001
0	12,888 (45.1)	2,020 (58.8)	10,868 (43.2)		12,282 (45.4)	1,923 (58.8)	10,359 (43.5)	
≥1	15,680 (54.9)	1,415 (41.2)	14,265 (56.8)		14,784 (54.6)	1,348 (41.2)	13,436(56.5)	≥1
Ever smokers (all participants), n (%):	13,389 (46.8)	1,722 (50.1)	11,667 (46.4)	< .001	13,186 (48.7)	1,668 (50.9)	11,518(48.4)	.006
Age at smoking initiation (current + former smokers), median (25th–75th percentiles)	17.0(15.0–19.0)	16.0(15.0–18.0)	17.0 (15.0–19.0)	.017	–	–	–	–
Current + former smokers who quit smoking, n (%):	7,627 (57.0)	910(52.8)	6,717 (57.6)	< .001	–	–	–	–
Cigarettes/week (current smokers),	42.0	52.5	40.0		70.0	70.0	70.0	
median (25th–75th percentiles)	(10.0–70.0)	(12.0–105)	(10.0–70.0)	< .001	(21.0–105)	(35.0–105)	(21.0–105)	.001

## Data Availability

Consent given by the participants does not open for storage of data on an individual level in repositories or journals. Researchers who want access to datasets for replication should apply to datatilgang@fhi.no. Access to datasets requires approval from the Regional Committee for Medical and Health Research Ethics in Norway and an agreement with MoBa. Source data of the GWAS on smoking initiation, age at smoking initiation, smoking cessation, and smoking intensity) are available in the Supplemental Tables of the article by Liu et al. (24) (https://www.nature.com/articles/s41588-018-0307-5#Sec14). Source data of the GWAS on education years are available in the Supplemental Tables of the article by Lee et al. (34) (https://www.nature.com/articles/s41588-018-0147-3#Sec34). Finally, source data of the GWAS on BMI are available in the GIANT Consortium website (https://portals.broadinstitute.org/collaboration/giant/index.php/GIANT_consortium_data_files#GWAS_Anthropometric_2015_BMI_Summary_Statistics).
