# Supplementary material for: Smoking and infertility: multivariable regression and Mendelian randomization analyses in the Norwegian Mother, Father and Child Cohort Study
Source: Fertil Steril. Author manuscript; Available in PMC 2022 Jul 6. (PMC7612999; doi:10.1016/j.fertnstert.2022.04.001)
Supplement: Supplementary file [file EMS146213-supplement-Supplementary_file.docx]

**SUPPLEMENTAL MATERIALS**

**SUPPLEMENTAL METHODS**

*Lifestyle-related covariates for sensitivity analyses*

Fiber

Fiber intake is a suitable proxy for diet quality during pregnancy (1, 2) that is available in MoBa mothers in the study database (1, 3). We estimated paternal fiber consumption from the questions interrogating about the intake frequency of foods with fiber in the baseline questionnaire (white bread, medium coarse-grain bread, full coarse-grain bread, crispbread, jam/jelly, Italian salad spread, vegetarian dishes, sausage/burgers, kebab, pizza, cooked vegetables, vegetable soup, salad, and fruit). We estimated fiber intake using this consumption frequency, the weights of an average serving of each food (4) and the average content of fiber in 100 g of each food according to the Norwegian Food Composition Tables (5), both described in **Supplemental Table 12**.

Physical activity

We estimated the energy expenditure in physical activity before pregnancy using information available in the baseline questionnaires. Women were asked about the average weekly frequency with which they performed 13 specific types of physical activity (walking, brisk walking, running/jogging, cycling, weight training, aerobics adapted to pregnant women, mid-intensity aerobics, high-intensity aerobics, dancing, skiing, ball sports, swimming, riding) before pregnancy. We estimated the energy expenditure as physical activity in metabolic equivalents of task (METs)-minute per week using these weekly frequencies, the average duration of an exercise session, and the average expenditure per minute in each exercise in METs (6), as described in **Supplemental Table 13**. In parallel, fathers were asked about the average weekly time they performed light physical activity (activities that did not imply sweating or gasp) and heavy physical activity (implied sweating or gasping for breath). We estimated their METs-min/week assuming an average energy expenditure of 4.5 and 8 METs-min for light and heavy physical activity, respectively (6).

Caffeine consumption

Regarding caffeine consumption, women were asked to report the number of daily cups (125 mL), mugs/glasses (250 mL), small bottles (500 mL) and large plastic bottles (1,500 mL) of caffeinated beverages (filter coffee, boiled coffee, instant coffee, tea, sugar-sweetened coke, and artificially sweetened coke) they consumed before pregnancy. Men were asked about their intake frequency of caffeinated drinks in the baseline questionnaire (the same in women plus energy drinks). Information on decaffeinated coffee, non-caffeinated tea (e.g., other herbal tea) and non-caffeinated fizzy drinks was also available but excluded for our estimations. We calculated the caffeine intake in mg per day using the consumption frequencies, the volume of an average serving, and the caffeine content per 100 mL of each drink (57 mg in filter coffee, 57 mg in boiled coffee, 40 mg in instant coffee, 16 mg in tea, 12 mg in coke, and 80 mL in energy drinks) (7).

Alcohol intake

Parents reported the frequency with which they drank alcohol prior to pregnancy (women: three months before; men: six months before) and the units of alcohol ingested whenever they drank (<1, 1-2, 3-4, 5-6, 7-9, ≥10). Fathers were asked to differentiate the units of alcohol they drank in weekdays and during the weekend; thus, we calculated a weighted average: [(units in weekdays × 5) + (units in weekends × 2)]/7. We computed the estimated units of alcohol consumption per week by multiplying the intake frequency per the ingested units (8). As there was a particularly high rate of missing values in the average units of alcohol ingested by fathers each time they drank, we finally used the drinking frequency as a covariate.

Occupational toxicants

Parents reported whether they were exposed to occupation-related toxicants in the immediate months before the pregnancy. In particular, they were interrogated if they had been exposed (yes/no) to lead (vapors, dust, particles or alloys), chromium, arsenic, cadmium, petrol/gasoline or exhaust fumes (not filling their own car), mercury vapors, amalgams (not as a patient), industrial dyes or inks, motor oil, lubricating oil, photographic chemicals, welding-related substances, soldering-related substances, formaldehyde, chemotherapy (not as a patient), and nitrous oxide (not as a patient).

*GWAS on infertility in the MoBa cohort*

We assessed the relationship between the genotyped SNPs in the MoBa cohort after the quality control process (9) and infertility by logistic regressions, in women and men separately. Any participant reporting a time-to-pregnancy ≥12 months or having undergone assisted reproductive technologies in any of their pregnancies was considered infertile. The MoBa Genetics infrastructure is a collaborative research initiative arranged in five genotype batches of parent-offspring trios (9, 10). We evaluated the association between the genotyped SNPs and infertility in each batch using Plink v1.9 (11) and lastly performed a sample size-weighted meta-analysis (random effects model) using the GWAMA software (12).

**SUPPLEMENTAL RESULTS**

*Robustness of genetic instruments for education years and body mass index*

The genetic instrument of education years was robust. Each one-unit increase in the GRS was associated with an increase of 0.028 education years in women (95% CI 0.026 to 0.030, *p* < 0.001, 4.11% of variance explained, *F*-statistic = 956) and 0.031 education years in men (95% CI 0.028 to 0.033, *p* < 0.001, 3.92% of variance explained, *F*-statistic = 837).

The genetic instrument of body mass index was also robust. In this case, each one-unit increase in the GRS was linked to an increase in body mass index of 0.044 kg/m^2^ in women (95% CI 0.041 to 0.046, *p* < 0.001, 5.65% of variance explained, *F*-statistic = 1,196) and 0.033 kg/m^2^ in men (95% CI 0.031 to 0.035, *p* < 0.001, 5.14% of variance, *F*-statistic = 1,036).

**SUPPLEMENTAL TABLES**

**Supplemental Table 1.** SNPs included in one-sample and two-sample MR analyses.

| **SNPs of smoking initiation** | | | | | | | | | | |
| --- | --- | --- | --- | --- | --- | --- | --- | --- | --- | --- |
| RSID | Chromosome | Position | One-sample  MR | Two-sample  MR | Reference  allele | Effect  allele | Effect allele  frequency | Beta | Standard  error | *p*-value |
| rs12130857 | 1 | 7791461 | Yes | Yes | G | A | 0.325 | -0.01800 | 0.00272 | 3.65E-11 |
| rs301807 | 1 | 8484823 | Yes | Yes | A | G | 0.570 | 0.01801 | 0.00257 | 2.50E-12 |
| rs3820277 | 1 | 18436657 | Yes | Yes | G | T | 0.526 | -0.01884 | 0.00255 | 1.57E-13 |
| rs1889571 | 1 | 32195819 | Yes | Yes | T | G | 0.131 | 0.02218 | 0.00378 | 4.19E-09 |
| rs10914684 | 1 | 33795572 | Yes | Yes | G | A | 0.324 | -0.01580 | 0.00272 | 6.32E-09 |
| rs2637869 | 1 | 38757237 | Yes | Yes | G | A | 0.297 | 0.01822 | 0.00279 | 6.54E-11 |
| rs12755632 | 1 | 41776623 | Yes | Yes | A | G | 0.316 | -0.01541 | 0.00274 | 1.93E-08 |
| rs951740 | 1 | 44011737 | Yes | Yes | G | A | 0.625 | 0.02954 | 0.00263 | 3.82E-29 |
| rs925524 | 1 | 46496709 | Yes | Yes | A | G | 0.710 | 0.01556 | 0.00281 | 2.94E-08 |
| rs12022778 | 1 | 50603995 | Yes | Yes | A | C | 0.202 | 0.02682 | 0.00318 | 3.18E-17 |
| rs11587399 | 1 | 50861071 | Yes | No | A | T | 0.221 | -0.01780 | 0.00308 | 7.25E-09 |
| rs4912332 | 1 | 58815243 | Yes | Yes | C | T | 0.491 | 0.01412 | 0.00255 | 2.94E-08 |
| rs1937443 | 1 | 66469643 | Yes | No | C | G | 0.563 | 0.02044 | 0.00257 | 1.79E-15 |
| rs1022528 | 1 | 71490122 | Yes | Yes | G | A | 0.344 | 0.01740 | 0.00268 | 8.48E-11 |
| rs12740789 | 1 | 72752073 | Yes | Yes | G | A | 0.178 | -0.02850 | 0.00333 | 1.18E-17 |
| rs80054503 | 1 | 72900406 | No | No | T | C | 0.116 | -0.02414 | 0.00407 | 3.10E-09 |
| rs10789369 | 1 | 73824909 | Yes | Yes | A | G | 0.615 | -0.02345 | 0.00262 | 3.39E-19 |
| rs1514176 | 1 | 74991596 | Yes | Yes | G | A | 0.580 | -0.01930 | 0.00258 | 7.67E-14 |
| rs10873871 | 1 | 76689019 | Yes | Yes | A | G | 0.207 | 0.01745 | 0.00314 | 2.82E-08 |
| rs11162019 | 1 | 87913176 | Yes | Yes | C | T | 0.363 | -0.01549 | 0.00265 | 5.06E-09 |
| rs1008078 | 1 | 91189731 | Yes | Yes | C | T | 0.402 | 0.02282 | 0.00260 | 1.63E-18 |
| rs1935571 | 1 | 96414335 | Yes | Yes | T | G | 0.480 | -0.01572 | 0.00255 | 6.99E-10 |
| rs12027999 | 1 | 154206358 | Yes | Yes | T | C | 0.120 | -0.02436 | 0.00392 | 5.33E-10 |
| rs45444697 | 1 | 155034632 | Yes | No | C | G | 0.212 | 0.01969 | 0.00312 | 2.72E-10 |
| rs2901785 | 1 | 174104743 | Yes | Yes | G | A | 0.446 | -0.01731 | 0.00256 | 1.47E-11 |
| rs147052174 | 1 | 179783167 | Yes | Yes | G | T | 0.017 | 0.06231 | 0.00983 | 2.30E-10 |
| rs35656245 | 1 | 190957480 | Yes | Yes | G | A | 0.276 | 0.01595 | 0.00285 | 2.23E-08 |
| rs12739243 | 1 | 210302043 | Yes | Yes | T | C | 0.221 | -0.02125 | 0.00307 | 4.45E-12 |
| rs12563365 | 1 | 236872829 | Yes | Yes | G | A | 0.556 | 0.01656 | 0.00256 | 1.05E-10 |
| rs876793 | 1 | 237852083 | Yes | Yes | T | C | 0.349 | -0.01792 | 0.00274 | 5.69E-11 |
| rs114976176 | 2 | 264621 | Yes | Yes | A | C | 0.352 | -0.01551 | 0.00267 | 6.04E-09 |
| rs62106258 | 2 | 417167 | Yes | Yes | T | C | 0.047 | -0.04550 | 0.00600 | 3.33E-14 |
| rs6731872 | 2 | 624205 | Yes | Yes | T | G | 0.826 | 0.03160 | 0.00336 | 5.35E-21 |
| rs1022376 | 2 | 22067213 | Yes | Yes | T | C | 0.516 | -0.01474 | 0.00261 | 1.66E-08 |
| rs61533748 | 2 | 22582968 | Yes | Yes | T | C | 0.384 | 0.01744 | 0.00262 | 2.82E-11 |
| rs72790288 | 2 | 29513404 | Yes | Yes | G | A | 0.028 | -0.04553 | 0.00770 | 3.28E-09 |
| rs2710634 | 2 | 32808804 | Yes | Yes | T | C | 0.521 | -0.01776 | 0.00255 | 3.36E-12 |
| rs62137126 | 2 | 44250149 | Yes | Yes | A | G | 0.121 | -0.02369 | 0.00391 | 1.31E-09 |
| rs1004787 | 2 | 45159091 | Yes | Yes | G | A | 0.552 | 0.02841 | 0.00256 | 1.11E-28 |
| rs7598402 | 2 | 50735943 | Yes | No | C | G | 0.492 | -0.01473 | 0.00255 | 7.38E-09 |
| rs10490159 | 2 | 51341259 | Yes | Yes | C | T | 0.394 | 0.01724 | 0.00261 | 3.86E-11 |
| rs1518393 | 2 | 58171220 | Yes | Yes | A | C | 0.619 | 0.01686 | 0.00262 | 1.30E-10 |
| rs17616642 | 2 | 59022210 | Yes | Yes | A | G | 0.247 | -0.01656 | 0.00295 | 2.10E-08 |
| rs6730325 | 2 | 59315828 | Yes | Yes | G | A | 0.610 | -0.01464 | 0.00261 | 2.10E-08 |
| rs2539706 | 2 | 59819545 | Yes | Yes | G | A | 0.530 | 0.01625 | 0.00255 | 1.95E-10 |
| rs7585579 | 2 | 60024857 | Yes | No | C | G | 0.499 | 0.02040 | 0.00261 | 5.48E-15 |
| rs1863161 | 2 | 60139524 | Yes | Yes | G | A | 0.561 | 0.01534 | 0.00257 | 2.34E-09 |
| rs359247 | 2 | 60477052 | Yes | No | A | T | 0.639 | 0.02203 | 0.00265 | 9.89E-17 |
| rs62180324 | 2 | 63416606 | Yes | Yes | G | A | 0.212 | -0.01952 | 0.00312 | 3.91E-10 |
| rs6750107 | 2 | 80748807 | Yes | Yes | G | A | 0.387 | 0.01457 | 0.00262 | 2.60E-08 |
| rs12714017 | 2 | 80999398 | Yes | Yes | T | C | 0.511 | 0.01540 | 0.00261 | 3.65E-09 |
| rs56208390 | 2 | 83247997 | Yes | Yes | A | G | 0.123 | 0.02156 | 0.00388 | 2.68E-08 |
| rs11692435 | 2 | 98275354 | Yes | Yes | G | A | 0.085 | 0.02505 | 0.00458 | 4.47E-08 |
| rs13392222 | 2 | 100672408 | Yes | Yes | A | C | 0.139 | -0.02344 | 0.00368 | 1.93E-10 |
| rs1901477 | 2 | 104126983 | Yes | Yes | A | G | 0.511 | 0.03044 | 0.00261 | 2.07E-31 |
| rs11889814 | 2 | 104432494 | Yes | Yes | A | C | 0.128 | -0.02103 | 0.00381 | 3.44E-08 |
| rs3811038 | 2 | 113240183 | Yes | Yes | T | C | 0.279 | 0.01914 | 0.00284 | 1.58E-11 |
| rs75210106 | 2 | 113246436 | Yes | Yes | C | T | 0.177 | -0.01866 | 0.00334 | 2.33E-08 |
| rs34399632 | 2 | 137571174 | Yes | Yes | A | G | 0.232 | 0.01935 | 0.00302 | 1.46E-10 |
| rs74697736 | 2 | 145412271 | Yes | Yes | G | A | 0.287 | 0.02230 | 0.00282 | 2.43E-15 |
| rs6756212 | 2 | 146140132 | Yes | Yes | C | T | 0.535 | -0.03389 | 0.00255 | 3.49E-40 |
| rs3076896 | 2 | 146283610 | No | No | G | A | 0.390 | 0.02265 | 0.00275 | 1.99E-16 |
| rs16826827 | 2 | 147825689 | Yes | Yes | T | C | 0.124 | -0.02221 | 0.00387 | 9.17E-09 |
| rs1445649 | 2 | 155682556 | Yes | Yes | T | C | 0.538 | 0.02057 | 0.00256 | 8.48E-16 |
| rs1722666 | 2 | 161816880 | Yes | Yes | C | T | 0.732 | 0.01609 | 0.00288 | 2.17E-08 |
| rs11678980 | 2 | 162101261 | Yes | Yes | G | A | 0.450 | 0.01767 | 0.00256 | 5.19E-12 |
| rs12474587 | 2 | 162802993 | Yes | Yes | G | T | 0.429 | 0.02423 | 0.00257 | 4.83E-21 |
| rs357304 | 2 | 164862639 | Yes | Yes | T | C | 0.727 | 0.01668 | 0.00286 | 5.40E-09 |
| rs13007361 | 2 | 166250244 | No | No | G | A | 0.208 | 0.01753 | 0.00314 | 2.29E-08 |
| rs7600835 | 2 | 172521827 | Yes | Yes | G | A | 0.342 | -0.01512 | 0.00269 | 1.80E-08 |
| rs6750529 | 2 | 182027603 | Yes | Yes | C | T | 0.744 | 0.01991 | 0.00292 | 9.26E-12 |
| rs17229285 | 2 | 199523122 | Yes | Yes | C | T | 0.505 | -0.01548 | 0.00255 | 1.27E-09 |
| rs3115418 | 2 | 200936399 | Yes | Yes | T | C | 0.454 | -0.01422 | 0.00256 | 2.79E-08 |
| rs62193862 | 2 | 202843875 | Yes | Yes | G | A | 0.100 | 0.02385 | 0.00425 | 1.99E-08 |
| rs4674916 | 2 | 225365635 | Yes | Yes | C | A | 0.328 | -0.01803 | 0.00271 | 3.06E-11 |
| rs4674993 | 2 | 226332033 | Yes | Yes | A | G | 0.200 | -0.02401 | 0.00319 | 4.85E-14 |
| rs11713899 | 3 | 2365026 | Yes | Yes | A | C | 0.171 | 0.01872 | 0.00338 | 3.15E-08 |
| rs748832 | 3 | 16851202 | Yes | Yes | A | G | 0.371 | 0.01721 | 0.00264 | 6.60E-11 |
| rs10446419 | 3 | 25725501 | Yes | Yes | A | G | 0.207 | -0.01956 | 0.00314 | 5.05E-10 |
| rs13319205 | 3 | 47800216 | Yes | No | T | A | 0.290 | 0.01654 | 0.00281 | 3.77E-09 |
| rs3172494 | 3 | 48731487 | Yes | Yes | G | T | 0.115 | -0.02913 | 0.00400 | 3.40E-13 |
| rs2526390 | 3 | 50192760 | Yes | Yes | C | T | 0.334 | 0.02047 | 0.00270 | 3.62E-14 |
| rs2276825 | 3 | 52886605 | Yes | Yes | T | C | 0.245 | 0.01888 | 0.00296 | 1.89E-10 |
| rs2306866 | 3 | 53766212 | Yes | No | A | T | 0.614 | -0.01668 | 0.00262 | 1.89E-10 |
| rs73831818 | 3 | 55988394 | Yes | Yes | A | G | 0.057 | 0.03204 | 0.00550 | 5.46E-09 |
| rs1910236 | 3 | 59434420 | Yes | Yes | G | A | 0.469 | 0.01464 | 0.00255 | 9.91E-09 |
| rs7640107 | 3 | 59966156 | Yes | Yes | C | T | 0.431 | -0.01419 | 0.00257 | 3.46E-08 |
| rs2734390 | 3 | 60459291 | Yes | Yes | A | G | 0.372 | 0.01477 | 0.00264 | 2.09E-08 |
| rs221988 | 3 | 64234307 | Yes | Yes | A | C | 0.384 | -0.01487 | 0.00262 | 1.43E-08 |
| rs2196356 | 3 | 70890288 | Yes | No | G | C | 0.289 | -0.01877 | 0.00281 | 2.45E-11 |
| rs11128203 | 3 | 71064431 | Yes | No | T | A | 0.530 | 0.02041 | 0.00255 | 1.29E-15 |
| rs62246017 | 3 | 71483084 | Yes | Yes | G | A | 0.323 | -0.01617 | 0.00273 | 3.03E-09 |
| rs4543050 | 3 | 74954560 | Yes | No | A | T | 0.816 | 0.02220 | 0.00329 | 1.45E-11 |
| rs6782116 | 3 | 77176032 | Yes | Yes | C | T | 0.415 | -0.01465 | 0.00259 | 1.46E-08 |
| rs13066050 | 3 | 81325861 | Yes | Yes | C | T | 0.208 | 0.01883 | 0.00314 | 1.93E-09 |
| rs12633090 | 3 | 83241365 | Yes | No | G | C | 0.182 | -0.02302 | 0.00330 | 3.16E-12 |
| rs1549979 | 3 | 85460131 | Yes | Yes | C | T | 0.615 | -0.02452 | 0.00262 | 8.80E-21 |
| rs74664784 | 3 | 85475292 | No | No | T | C | 0.376 | -0.01991 | 0.00279 | 9.34E-13 |
| rs57153235 | 3 | 85902536 | Yes | Yes | T | G | 0.318 | -0.01938 | 0.00274 | 1.56E-12 |
| rs6437769 | 3 | 107997514 | Yes | Yes | C | T | 0.581 | 0.01421 | 0.00258 | 3.74E-08 |
| rs9288999 | 3 | 114147927 | Yes | Yes | G | A | 0.735 | 0.01744 | 0.00289 | 1.50E-09 |
| rs6438436 | 3 | 117822149 | Yes | Yes | C | T | 0.816 | 0.02474 | 0.00329 | 5.33E-14 |
| rs12053870 | 3 | 118302515 | No | No | T | G | 0.542 | 0.01562 | 0.00256 | 1.02E-09 |
| rs9826984 | 3 | 131945722 | Yes | Yes | G | A | 0.542 | -0.01405 | 0.00256 | 3.87E-08 |
| rs2279829 | 3 | 147106319 | Yes | Yes | C | T | 0.216 | -0.01738 | 0.00310 | 2.05E-08 |
| rs2319545 | 3 | 147719648 | Yes | Yes | C | A | 0.149 | 0.02324 | 0.00358 | 8.30E-11 |
| rs10935779 | 3 | 149543102 | Yes | Yes | C | T | 0.415 | -0.01433 | 0.00259 | 2.95E-08 |
| rs963354 | 3 | 157393770 | Yes | Yes | C | A | 0.687 | 0.01505 | 0.00275 | 4.21E-08 |
| rs1714521 | 3 | 158284861 | Yes | Yes | A | C | 0.411 | -0.01630 | 0.00259 | 3.07E-10 |
| rs1449012 | 3 | 159048333 | Yes | Yes | C | T | 0.463 | -0.01537 | 0.00256 | 1.77E-09 |
| rs9850597 | 3 | 161761866 | Yes | Yes | G | A | 0.816 | -0.01857 | 0.00329 | 1.65E-08 |
| rs1187820 | 3 | 173072584 | Yes | Yes | C | T | 0.439 | -0.01427 | 0.00257 | 2.69E-08 |
| rs16828799 | 3 | 173353739 | Yes | Yes | G | T | 0.156 | 0.01977 | 0.00351 | 1.83E-08 |
| rs9841807 | 3 | 175718927 | No | No | C | T | 0.273 | 0.01625 | 0.00286 | 1.35E-08 |
| rs7631379 | 3 | 181409057 | Yes | Yes | T | C | 0.206 | 0.02080 | 0.00315 | 3.94E-11 |
| rs4140932 | 4 | 15458598 | Yes | No | T | A | 0.431 | -0.01404 | 0.00257 | 4.89E-08 |
| rs12642744 | 4 | 28027176 | Yes | Yes | G | T | 0.744 | -0.01659 | 0.00299 | 2.82E-08 |
| rs59537158 | 4 | 28246049 | Yes | Yes | C | T | 0.214 | 0.02249 | 0.00311 | 4.62E-13 |
| rs1389171 | 4 | 28822284 | Yes | No | T | A | 0.241 | -0.01747 | 0.00298 | 4.45E-09 |
| rs55944129 | 4 | 29082156 | Yes | Yes | T | C | 0.267 | -0.01757 | 0.00288 | 1.06E-09 |
| rs58400863 | 4 | 31184484 | Yes | Yes | G | A | 0.347 | -0.02017 | 0.00268 | 4.89E-14 |
| rs7657022 | 4 | 35501032 | Yes | Yes | A | G | 0.489 | 0.01829 | 0.00255 | 7.34E-13 |
| rs55900829 | 4 | 35514712 | No | No | A | T | 0.335 | 0.01913 | 0.00278 | 5.63E-12 |
| rs112725451 | 4 | 68017710 | Yes | Yes | C | T | 0.169 | 0.02609 | 0.00340 | 1.65E-14 |
| rs1160685 | 4 | 94052854 | Yes | No | C | G | 0.450 | 0.01530 | 0.00256 | 2.31E-09 |
| rs1435479 | 4 | 94550450 | Yes | Yes | G | T | 0.287 | 0.01639 | 0.00282 | 5.68E-09 |
| rs3934797 | 4 | 112467612 | No | No | G | A | 0.182 | -0.02130 | 0.00330 | 1.12E-10 |
| rs71602617 | 4 | 136406155 | Yes | Yes | C | T | 0.216 | -0.01777 | 0.00317 | 2.10E-08 |
| rs7696257 | 4 | 137474783 | Yes | Yes | G | A | 0.366 | 0.01533 | 0.00264 | 6.78E-09 |
| rs13109980 | 4 | 140886963 | Yes | Yes | G | A | 0.326 | -0.02218 | 0.00272 | 3.37E-16 |
| rs1116690 | 4 | 143510148 | Yes | Yes | A | G | 0.742 | 0.01629 | 0.00291 | 2.16E-08 |
| rs13110073 | 4 | 147797913 | Yes | Yes | T | C | 0.395 | -0.02464 | 0.00261 | 3.24E-21 |
| rs28717373 | 4 | 147985231 | Yes | Yes | C | T | 0.356 | -0.01647 | 0.00266 | 6.16E-10 |
| rs62340589 | 4 | 176875795 | Yes | No | G | C | 0.201 | 0.01741 | 0.00318 | 4.31E-08 |
| rs12517438 | 5 | 30842054 | Yes | Yes | T | G | 0.538 | 0.01535 | 0.00256 | 1.89E-09 |
| rs35375873 | 5 | 43190647 | Yes | No | G | C | 0.110 | -0.02701 | 0.00407 | 3.29E-11 |
| rs986714 | 5 | 50821338 | Yes | No | A | T | 0.445 | -0.01603 | 0.00256 | 4.13E-10 |
| rs71592686 | 5 | 60121271 | Yes | Yes | T | C | 0.274 | 0.02074 | 0.00286 | 3.85E-13 |
| rs2028269 | 5 | 79308315 | Yes | Yes | G | A | 0.399 | 0.01616 | 0.00260 | 5.19E-10 |
| rs6874731 | 5 | 80263865 | Yes | Yes | T | G | 0.484 | 0.01532 | 0.00255 | 1.83E-09 |
| rs6452785 | 5 | 87685500 | Yes | Yes | C | T | 0.474 | -0.02688 | 0.00255 | 4.69E-26 |
| rs10805858 | 5 | 88873832 | Yes | No | A | T | 0.335 | 0.01812 | 0.00270 | 1.88E-11 |
| rs181508347 | 5 | 91366274 | Yes | Yes | T | G | 0.010 | 0.08108 | 0.01303 | 4.95E-10 |
| rs42417 | 5 | 94198290 | Yes | Yes | C | T | 0.691 | 0.01693 | 0.00276 | 8.27E-10 |
| rs72780746 | 5 | 103929588 | Yes | Yes | T | C | 0.173 | -0.02576 | 0.00337 | 2.05E-14 |
| rs10060196 | 5 | 106455988 | Yes | Yes | C | A | 0.581 | 0.01831 | 0.00258 | 1.29E-12 |
| rs72789626 | 5 | 106825618 | Yes | No | T | A | 0.136 | -0.02564 | 0.00372 | 5.13E-12 |
| rs17165769 | 5 | 107365642 | Yes | Yes | A | G | 0.395 | 0.01594 | 0.00261 | 9.56E-10 |
| rs329124 | 5 | 133865452 | Yes | Yes | A | G | 0.428 | -0.01639 | 0.00257 | 1.96E-10 |
| rs1385108 | 5 | 154839646 | Yes | Yes | C | T | 0.239 | 0.01870 | 0.00299 | 3.84E-10 |
| rs1173461 | 5 | 157707571 | Yes | Yes | C | T | 0.327 | 0.01661 | 0.00272 | 9.51E-10 |
| rs11956866 | 5 | 161018271 | Yes | Yes | T | G | 0.567 | -0.01484 | 0.00257 | 7.82E-09 |
| rs3909281 | 5 | 165096435 | Yes | Yes | T | G | 0.536 | 0.02107 | 0.00255 | 1.62E-16 |
| rs3843905 | 5 | 165427280 | Yes | Yes | C | T | 0.403 | -0.01515 | 0.00260 | 5.41E-09 |
| rs79476395 | 5 | 166063680 | Yes | Yes | A | G | 0.073 | 0.03337 | 0.00491 | 1.04E-11 |
| rs6890961 | 5 | 166778503 | No | No | C | T | 0.624 | -0.01931 | 0.00263 | 2.13E-13 |
| rs4044321 | 5 | 166989513 | Yes | Yes | A | G | 0.644 | -0.02264 | 0.00266 | 1.75E-17 |
| rs2173019 | 5 | 167614971 | Yes | No | T | A | 0.177 | 0.02821 | 0.00334 | 2.98E-17 |
| rs10042827 | 5 | 170299916 | Yes | No | T | C | 0.681 | 0.01672 | 0.00273 | 9.41E-10 |
| rs359431 | 5 | 173288534 | Yes | Yes | C | T | 0.560 | -0.01420 | 0.00257 | 3.16E-08 |
| rs1059490 | 6 | 26171250 | Yes | Yes | T | C | 0.367 | -0.01859 | 0.00265 | 2.16E-12 |
| rs6932350 | 6 | 26571629 | Yes | No | T | A | 0.455 | 0.01497 | 0.00256 | 5.13E-09 |
| rs1150668 | 6 | 28129789 | Yes | Yes | T | G | 0.419 | -0.01851 | 0.00259 | 8.54E-13 |
| rs1632941 | 6 | 29796685 | No | No | T | C | 0.460 | -0.01581 | 0.00256 | 6.67E-10 |
| rs3218116 | 6 | 41901763 | Yes | Yes | C | T | 0.256 | -0.01984 | 0.00292 | 1.05E-11 |
| rs160631 | 6 | 52895230 | Yes | Yes | T | G | 0.731 | -0.01726 | 0.00287 | 1.87E-09 |
| rs7743165 | 6 | 67521222 | Yes | Yes | T | G | 0.495 | 0.01926 | 0.00255 | 4.15E-14 |
| rs79180767 | 6 | 67540984 | No | No | C | T | 0.253 | 0.02009 | 0.00293 | 7.00E-12 |
| rs10945141 | 6 | 69470709 | Yes | Yes | G | A | 0.263 | 0.01814 | 0.00289 | 3.59E-10 |
| rs17554906 | 6 | 92226609 | Yes | No | G | C | 0.444 | 0.01418 | 0.00256 | 3.14E-08 |
| rs619087 | 6 | 94175279 | Yes | Yes | A | G | 0.422 | 0.01427 | 0.00258 | 3.10E-08 |
| rs6568832 | 6 | 97702876 | Yes | Yes | G | A | 0.754 | 0.01887 | 0.00296 | 1.74E-10 |
| rs12195240 | 6 | 98636905 | Yes | Yes | G | A | 0.285 | 0.02491 | 0.00282 | 1.08E-18 |
| rs6936160 | 6 | 100347745 | Yes | Yes | C | T | 0.698 | 0.02011 | 0.00277 | 4.20E-13 |
| rs12530388 | 6 | 101329173 | Yes | Yes | A | C | 0.511 | -0.01836 | 0.00255 | 5.83E-13 |
| rs3800227 | 6 | 108994161 | Yes | Yes | A | G | 0.742 | 0.01718 | 0.00291 | 3.64E-09 |
| rs118202 | 6 | 111658371 | Yes | Yes | G | T | 0.812 | -0.03675 | 0.00326 | 1.90E-29 |
| rs73008357 | 6 | 156431856 | Yes | Yes | A | C | 0.121 | -0.02231 | 0.00400 | 2.44E-08 |
| rs9331343 | 6 | 157738258 | No | No | T | C | 0.568 | -0.01413 | 0.00257 | 3.90E-08 |
| rs10698713 | 6 | 158882320 | Yes | Yes | G | A | 0.054 | -0.03352 | 0.00562 | 2.38E-09 |
| rs1737329 | 6 | 163807748 | Yes | No | C | G | 0.742 | 0.01703 | 0.00291 | 5.08E-09 |
| rs10272990 | 7 | 1703675 | Yes | Yes | T | C | 0.328 | -0.02092 | 0.00271 | 1.27E-14 |
| rs6948707 | 7 | 1870794 | Yes | Yes | T | G | 0.419 | 0.02435 | 0.00258 | 4.24E-21 |
| rs10259715 | 7 | 3329967 | No | No | T | A | 0.210 | -0.01867 | 0.00322 | 6.42E-09 |
| rs13237637 | 7 | 3503207 | Yes | No | G | C | 0.485 | -0.02368 | 0.00255 | 1.54E-20 |
| rs79631993 | 7 | 69432311 | No | No | A | C | 0.216 | -0.01703 | 0.00309 | 3.67E-08 |
| rs7809303 | 7 | 69484366 | Yes | Yes | G | A | 0.325 | -0.02142 | 0.00272 | 3.48E-15 |
| rs7802996 | 7 | 77771983 | Yes | Yes | C | T | 0.166 | -0.02088 | 0.00342 | 1.06E-09 |
| rs1030015 | 7 | 78139581 | Yes | Yes | G | T | 0.520 | 0.01429 | 0.00255 | 2.15E-08 |
| rs4727189 | 7 | 88442568 | Yes | Yes | T | C | 0.344 | 0.01486 | 0.00268 | 3.00E-08 |
| rs76841737 | 7 | 91281409 | Yes | No | C | G | 0.103 | -0.02315 | 0.00419 | 3.26E-08 |
| rs11768481 | 7 | 96629103 | Yes | Yes | C | A | 0.340 | -0.01856 | 0.00269 | 5.23E-12 |
| rs1799068 | 7 | 97707069 | Yes | Yes | G | T | 0.379 | 0.01661 | 0.00263 | 2.59E-10 |
| rs13437771 | 7 | 99071478 | Yes | Yes | A | G | 0.155 | -0.02711 | 0.00352 | 1.39E-14 |
| rs11766326 | 7 | 111100585 | Yes | Yes | T | C | 0.506 | -0.01754 | 0.00261 | 1.79E-11 |
| rs6968380 | 7 | 114940159 | Yes | Yes | G | A | 0.681 | -0.02342 | 0.00273 | 1.05E-17 |
| rs112913817 | 7 | 115077394 | No | No | A | G | 0.011 | 0.07806 | 0.01204 | 9.28E-11 |
| rs10233018 | 7 | 117523709 | Yes | Yes | A | G | 0.516 | 0.02461 | 0.00255 | 4.77E-22 |
| rs10953957 | 7 | 121954709 | Yes | Yes | G | A | 0.386 | 0.01441 | 0.00262 | 3.66E-08 |
| rs77283305 | 7 | 132593831 | Yes | Yes | G | A | 0.306 | -0.01520 | 0.00277 | 3.91E-08 |
| rs10279261 | 7 | 133589846 | Yes | Yes | G | A | 0.618 | -0.01887 | 0.00262 | 6.05E-13 |
| rs1561112 | 7 | 133840652 | Yes | Yes | T | C | 0.413 | -0.01524 | 0.00259 | 3.84E-09 |
| rs2952251 | 8 | 10143164 | Yes | Yes | A | G | 0.744 | 0.01641 | 0.00300 | 4.24E-08 |
| rs4326350 | 8 | 10763655 | Yes | No | C | G | 0.493 | -0.01761 | 0.00255 | 5.16E-12 |
| rs11780471 | 8 | 27344719 | Yes | Yes | G | A | 0.063 | -0.03868 | 0.00524 | 1.57E-13 |
| rs11783093 | 8 | 27425349 | Yes | Yes | C | T | 0.158 | -0.04712 | 0.00349 | 2.07E-41 |
| rs1565735 | 8 | 27426077 | Yes | No | T | A | 0.204 | -0.01916 | 0.00316 | 1.33E-09 |
| rs7836565 | 8 | 52569449 | Yes | Yes | C | T | 0.718 | -0.01551 | 0.00283 | 4.36E-08 |
| rs13261666 | 8 | 59814666 | Yes | Yes | G | T | 0.517 | -0.01999 | 0.00255 | 4.36E-15 |
| rs3850736 | 8 | 64912021 | Yes | No | C | G | 0.474 | 0.01913 | 0.00255 | 6.43E-14 |
| rs2063976 | 8 | 91096366 | Yes | Yes | C | T | 0.665 | -0.02018 | 0.00270 | 7.45E-14 |
| rs6993429 | 8 | 92733282 | Yes | Yes | C | A | 0.453 | -0.01905 | 0.00256 | 9.87E-14 |
| rs6986430 | 8 | 93048104 | Yes | Yes | T | C | 0.222 | -0.02434 | 0.00306 | 1.99E-15 |
| rs9987376 | 8 | 93190014 | Yes | Yes | T | G | 0.574 | -0.02047 | 0.00258 | 2.01E-15 |
| rs290601 | 8 | 115374642 | Yes | Yes | C | T | 0.274 | 0.01631 | 0.00286 | 1.14E-08 |
| rs3847244 | 9 | 3025368 | Yes | Yes | C | T | 0.470 | 0.01867 | 0.00255 | 2.60E-13 |
| rs11791671 | 9 | 3398679 | Yes | Yes | C | T | 0.067 | 0.02785 | 0.00508 | 4.24E-08 |
| rs7024924 | 9 | 8282399 | Yes | Yes | T | C | 0.174 | 0.01889 | 0.00336 | 1.90E-08 |
| rs6474609 | 9 | 10981069 | Yes | No | T | A | 0.587 | -0.01559 | 0.00259 | 1.71E-09 |
| rs1931431 | 9 | 11161799 | Yes | No | G | C | 0.478 | 0.01823 | 0.00255 | 8.56E-13 |
| rs7867822 | 9 | 20676454 | Yes | Yes | A | G | 0.673 | -0.01510 | 0.00272 | 2.76E-08 |
| rs10966092 | 9 | 23831658 | Yes | Yes | T | C | 0.267 | -0.02049 | 0.00288 | 1.12E-12 |
| rs10969352 | 9 | 29747488 | Yes | No | T | A | 0.500 | 0.01435 | 0.00255 | 1.82E-08 |
| rs4877285 | 9 | 81354129 | Yes | Yes | G | A | 0.668 | -0.01813 | 0.00271 | 2.10E-11 |
| rs1930371 | 9 | 81444104 | Yes | Yes | C | T | 0.241 | -0.01724 | 0.00298 | 7.09E-09 |
| rs2378662 | 9 | 86707289 | Yes | Yes | G | A | 0.541 | 0.01521 | 0.00256 | 2.67E-09 |
| rs1927901 | 9 | 120519111 | Yes | Yes | T | C | 0.553 | -0.01418 | 0.00256 | 3.10E-08 |
| rs4837631 | 9 | 122061948 | Yes | Yes | C | T | 0.446 | -0.01536 | 0.00256 | 2.03E-09 |
| rs1759433 | 9 | 128073097 | Yes | Yes | G | A | 0.480 | 0.01536 | 0.00255 | 1.69E-09 |
| rs34553878 | 9 | 134334588 | Yes | Yes | A | G | 0.111 | 0.02467 | 0.00406 | 1.17E-09 |
| rs7026534 | 9 | 134907263 | Yes | Yes | T | G | 0.704 | -0.01660 | 0.00279 | 2.68E-09 |
| rs10858334 | 9 | 137989785 | Yes | No | C | G | 0.140 | 0.02287 | 0.00376 | 1.18E-09 |
| rs10905461 | 10 | 8803551 | Yes | Yes | T | C | 0.748 | -0.01639 | 0.00293 | 2.36E-08 |
| rs7920501 | 10 | 10043159 | Yes | No | T | A | 0.465 | -0.01552 | 0.00255 | 1.25E-09 |
| rs1291821 | 10 | 11133823 | Yes | Yes | A | G | 0.534 | 0.01449 | 0.00255 | 1.39E-08 |
| rs11258417 | 10 | 13533053 | Yes | Yes | C | T | 0.391 | -0.01451 | 0.00261 | 2.71E-08 |
| rs7072776 | 10 | 22032942 | Yes | Yes | A | G | 0.712 | -0.02197 | 0.00281 | 5.66E-15 |
| rs2796793 | 10 | 36634124 | Yes | Yes | G | A | 0.452 | 0.01448 | 0.00256 | 1.55E-08 |
| rs1733760 | 10 | 56698174 | Yes | Yes | T | C | 0.510 | 0.01477 | 0.00255 | 6.70E-09 |
| rs7921378 | 10 | 63674885 | Yes | No | G | C | 0.482 | -0.02331 | 0.00255 | 6.10E-20 |
| rs7901883 | 10 | 103186838 | Yes | Yes | G | A | 0.230 | -0.01926 | 0.00303 | 1.98E-10 |
| rs11594623 | 10 | 103960351 | Yes | Yes | T | C | 0.234 | 0.02744 | 0.00301 | 7.45E-20 |
| rs11191269 | 10 | 104120522 | Yes | No | C | G | 0.193 | 0.01764 | 0.00323 | 4.61E-08 |
| rs28408682 | 10 | 104403310 | Yes | Yes | A | G | 0.600 | 0.01667 | 0.00260 | 1.41E-10 |
| rs12244388 | 10 | 104640052 | Yes | Yes | G | A | 0.350 | 0.02582 | 0.00267 | 4.31E-22 |
| rs111842178 | 10 | 104852121 | No | No | A | G | 0.231 | 0.02245 | 0.00320 | 2.24E-12 |
| rs34970111 | 10 | 106078937 | Yes | Yes | C | T | 0.458 | -0.01456 | 0.00256 | 1.28E-08 |
| rs9787523 | 10 | 106460460 | Yes | Yes | T | C | 0.418 | -0.01563 | 0.00258 | 1.42E-09 |
| rs11192347 | 10 | 106929313 | Yes | Yes | G | A | 0.104 | -0.02645 | 0.00427 | 6.15E-10 |
| rs10885480 | 10 | 115378364 | Yes | Yes | T | C | 0.284 | -0.01868 | 0.00283 | 3.83E-11 |
| rs4752018 | 10 | 118678712 | Yes | Yes | C | A | 0.231 | 0.01885 | 0.00302 | 4.42E-10 |
| rs9423279 | 10 | 125680419 | Yes | No | C | G | 0.645 | -0.01858 | 0.00266 | 3.06E-12 |
| rs6265 | 11 | 27679916 | Yes | Yes | C | T | 0.188 | -0.02928 | 0.00326 | 2.81E-19 |
| rs4275621 | 11 | 28652996 | Yes | Yes | A | G | 0.382 | -0.02137 | 0.00262 | 3.76E-16 |
| rs62618693 | 11 | 32956492 | Yes | Yes | C | T | 0.043 | -0.03527 | 0.00629 | 2.09E-08 |
| rs2939756 | 11 | 41436297 | Yes | Yes | G | A | 0.480 | -0.01570 | 0.00255 | 7.45E-10 |
| rs1381775 | 11 | 42442826 | Yes | Yes | T | C | 0.712 | -0.01561 | 0.00281 | 2.79E-08 |
| rs2959084 | 11 | 46078656 | Yes | Yes | G | A | 0.705 | 0.01708 | 0.00279 | 9.82E-10 |
| rs3740977 | 11 | 46393574 | Yes | Yes | T | C | 0.167 | 0.01947 | 0.00342 | 1.17E-08 |
| rs61886926 | 11 | 64133552 | Yes | Yes | C | T | 0.384 | -0.01794 | 0.00262 | 7.30E-12 |
| rs61884449 | 11 | 64485193 | Yes | Yes | C | T | 0.149 | 0.01998 | 0.00358 | 2.32E-08 |
| rs644740 | 11 | 65561468 | Yes | Yes | C | T | 0.457 | -0.01408 | 0.00256 | 3.67E-08 |
| rs7943721 | 11 | 73309393 | Yes | Yes | G | A | 0.829 | -0.02121 | 0.00338 | 3.58E-10 |
| rs7929518 | 11 | 85980958 | Yes | Yes | A | G | 0.773 | 0.01924 | 0.00304 | 2.55E-10 |
| rs586699 | 11 | 92289734 | No | No | G | A | 0.543 | -0.01480 | 0.00256 | 7.29E-09 |
| rs76460663 | 11 | 111979741 | No | No | C | G | 0.041 | -0.04235 | 0.00642 | 4.15E-11 |
| rs2155646 | 11 | 112912811 | No | No | T | C | 0.400 | 0.03778 | 0.00260 | 9.44E-48 |
| rs78239456 | 11 | 112984491 | No | No | A | T | 0.377 | -0.01850 | 0.00271 | 9.37E-12 |
| rs1713676 | 11 | 113660576 | Yes | Yes | A | G | 0.523 | -0.01673 | 0.00255 | 5.38E-11 |
| rs238896 | 11 | 113994505 | Yes | Yes | G | A | 0.490 | -0.01687 | 0.00255 | 3.65E-11 |
| rs540860 | 11 | 121530888 | Yes | Yes | A | G | 0.543 | 0.01761 | 0.00256 | 5.75E-12 |
| rs1944689 | 11 | 121634334 | Yes | Yes | G | T | 0.786 | 0.01768 | 0.00311 | 1.27E-08 |
| rs1834306 | 11 | 122023187 | Yes | Yes | A | G | 0.579 | -0.01449 | 0.00258 | 1.96E-08 |
| rs1106363 | 11 | 131966264 | Yes | Yes | C | T | 0.345 | 0.01737 | 0.00268 | 9.20E-11 |
| rs2010921 | 11 | 132098205 | Yes | Yes | G | A | 0.311 | 0.01743 | 0.00275 | 2.47E-10 |
| rs11057005 | 12 | 16748721 | Yes | Yes | A | G | 0.441 | -0.01571 | 0.00257 | 9.12E-10 |
| rs13906 | 12 | 49952394 | Yes | Yes | C | T | 0.109 | -0.02453 | 0.00409 | 1.98E-09 |
| rs4759229 | 12 | 56474480 | Yes | Yes | A | G | 0.656 | 0.01557 | 0.00268 | 6.53E-09 |
| rs7969559 | 12 | 69655167 | Yes | Yes | A | G | 0.713 | -0.01702 | 0.00282 | 1.53E-09 |
| rs7134009 | 12 | 75263193 | Yes | Yes | T | C | 0.287 | -0.01580 | 0.00288 | 4.30E-08 |
| rs77215829 | 12 | 112618346 | Yes | Yes | A | C | 0.131 | -0.02404 | 0.00378 | 2.02E-10 |
| rs1109480 | 12 | 121083279 | Yes | Yes | G | A | 0.384 | -0.01669 | 0.00262 | 1.84E-10 |
| rs11611651 | 12 | 133380790 | Yes | Yes | G | A | 0.087 | 0.02711 | 0.00453 | 2.05E-09 |
| rs17197663 | 13 | 38172867 | Yes | Yes | G | A | 0.125 | -0.02159 | 0.00385 | 2.06E-08 |
| rs4264267 | 13 | 38359676 | Yes | Yes | C | T | 0.527 | 0.01479 | 0.00255 | 6.82E-09 |
| rs61959481 | 13 | 55834929 | Yes | Yes | G | A | 0.210 | -0.02034 | 0.00313 | 7.95E-11 |
| rs3098272 | 13 | 55931424 | Yes | Yes | A | C | 0.799 | -0.01781 | 0.00318 | 2.08E-08 |
| rs9538162 | 13 | 59265043 | Yes | Yes | T | C | 0.416 | 0.01738 | 0.00258 | 1.76E-11 |
| rs1413119 | 13 | 59339281 | Yes | Yes | C | T | 0.396 | -0.01526 | 0.00260 | 4.77E-09 |
| rs56367474 | 13 | 59454139 | Yes | Yes | C | T | 0.304 | -0.01730 | 0.00277 | 4.20E-10 |
| rs55786907 | 13 | 59871584 | Yes | Yes | A | G | 0.162 | 0.01945 | 0.00345 | 1.84E-08 |
| rs4886207 | 13 | 60705792 | Yes | Yes | T | C | 0.637 | -0.01625 | 0.00265 | 8.78E-10 |
| rs9540731 | 13 | 66949370 | Yes | Yes | C | T | 0.509 | -0.01773 | 0.00255 | 3.42E-12 |
| rs9545155 | 13 | 80191873 | Yes | Yes | T | C | 0.478 | -0.01607 | 0.00255 | 3.04E-10 |
| rs1772572 | 13 | 81191176 | Yes | Yes | C | A | 0.324 | -0.01687 | 0.00272 | 5.62E-10 |
| rs75674569 | 13 | 96823724 | Yes | Yes | G | A | 0.100 | -0.02534 | 0.00425 | 2.58E-09 |
| rs7333559 | 13 | 100546450 | Yes | Yes | G | A | 0.783 | -0.02321 | 0.00309 | 5.94E-14 |
| rs1108130 | 13 | 100648356 | Yes | No | T | A | 0.212 | 0.02394 | 0.00312 | 1.57E-14 |
| rs12855717 | 13 | 101252635 | Yes | Yes | C | T | 0.538 | 0.01552 | 0.00256 | 1.22E-09 |
| rs12878369 | 14 | 28346502 | Yes | No | C | A | 0.415 | 0.01744 | 0.00259 | 1.60E-11 |
| rs2145451 | 14 | 29316842 | Yes | No | T | C | 0.193 | -0.02005 | 0.00323 | 5.44E-10 |
| rs9323328 | 14 | 58653514 | Yes | No | A | G | 0.537 | -0.01424 | 0.00256 | 2.55E-08 |
| rs1811739 | 14 | 77529375 | Yes | No | G | A | 0.248 | 0.01827 | 0.00295 | 5.97E-10 |
| rs8005334 | 14 | 79563654 | Yes | No | T | G | 0.360 | 0.01667 | 0.00266 | 3.44E-10 |
| rs34940743 | 14 | 80102233 | Yes | No | A | G | 0.346 | 0.01592 | 0.00268 | 2.80E-09 |
| rs2925128 | 14 | 98362355 | Yes | No | C | T | 0.385 | 0.01682 | 0.00268 | 3.67E-10 |
| rs1381287 | 14 | 98597552 | Yes | No | C | T | 0.467 | 0.01802 | 0.00256 | 1.81E-12 |
| rs55913542 | 14 | 99693843 | Yes | No | G | T | 0.175 | 0.01856 | 0.00336 | 3.25E-08 |
| rs1435672 | 15 | 36399479 | Yes | Yes | T | C | 0.560 | 0.01411 | 0.00257 | 3.82E-08 |
| rs281296 | 15 | 47685010 | Yes | Yes | G | A | 0.357 | 0.02469 | 0.00266 | 1.59E-20 |
| rs1435741 | 15 | 47935843 | Yes | Yes | G | A | 0.433 | 0.01831 | 0.00257 | 1.09E-12 |
| rs56902655 | 15 | 63898709 | Yes | Yes | T | G | 0.136 | -0.02186 | 0.00372 | 4.09E-09 |
| rs2289791 | 15 | 67476952 | Yes | Yes | G | T | 0.247 | -0.01773 | 0.00295 | 2.01E-09 |
| rs60833441 | 15 | 74048768 | Yes | Yes | A | G | 0.461 | -0.01428 | 0.00256 | 2.28E-08 |
| rs62007780 | 15 | 78025464 | Yes | Yes | G | T | 0.416 | -0.01591 | 0.00258 | 7.48E-10 |
| rs12442563 | 15 | 83893243 | Yes | Yes | G | T | 0.223 | -0.02323 | 0.00306 | 3.13E-14 |
| rs4310804 | 15 | 96858409 | Yes | No | C | G | 0.247 | -0.01819 | 0.00295 | 7.55E-10 |
| rs8027457 | 15 | 99204101 | Yes | Yes | T | C | 0.511 | 0.01531 | 0.00255 | 1.88E-09 |
| rs1139897 | 16 | 720986 | Yes | Yes | G | A | 0.230 | -0.02409 | 0.00303 | 1.77E-15 |
| rs11076962 | 16 | 5811367 | Yes | Yes | T | C | 0.279 | 0.01830 | 0.00284 | 1.20E-10 |
| rs7192140 | 16 | 10173748 | Yes | Yes | T | C | 0.498 | -0.01688 | 0.00255 | 3.40E-11 |
| rs9922607 | 16 | 17570220 | Yes | Yes | C | T | 0.200 | -0.02216 | 0.00319 | 3.42E-12 |
| rs9941217 | 16 | 18050926 | Yes | No | C | G | 0.352 | -0.01856 | 0.00267 | 3.50E-12 |
| rs7188873 | 16 | 24727064 | Yes | Yes | A | G | 0.613 | 0.02030 | 0.00262 | 8.46E-15 |
| rs6497840 | 16 | 25351633 | Yes | Yes | G | A | 0.707 | 0.02277 | 0.00287 | 2.01E-15 |
| rs4785187 | 16 | 49766772 | Yes | Yes | G | A | 0.223 | 0.01998 | 0.00306 | 6.55E-11 |
| rs8050598 | 16 | 49891964 | Yes | Yes | C | T | 0.254 | 0.01867 | 0.00293 | 1.76E-10 |
| rs12918191 | 16 | 50945156 | Yes | Yes | A | G | 0.243 | -0.01973 | 0.00297 | 3.14E-11 |
| rs9302604 | 16 | 69576894 | Yes | Yes | A | G | 0.435 | 0.01871 | 0.00257 | 3.29E-13 |
| rs9936784 | 16 | 72230694 | Yes | Yes | T | G | 0.534 | 0.01399 | 0.00255 | 4.33E-08 |
| rs62052916 | 16 | 72574550 | Yes | No | A | T | 0.070 | -0.03191 | 0.00499 | 1.62E-10 |
| rs4788676 | 16 | 72950468 | Yes | Yes | T | C | 0.229 | -0.01775 | 0.00303 | 4.92E-09 |
| rs61537885 | 16 | 75620118 | No | No | T | C | 0.037 | -0.04006 | 0.00694 | 8.06E-09 |
| rs117657830 | 16 | 75766873 | Yes | Yes | A | G | 0.042 | -0.03776 | 0.00637 | 3.18E-09 |
| rs1050847 | 16 | 87443734 | Yes | Yes | C | T | 0.559 | -0.01483 | 0.00257 | 7.37E-09 |
| rs11642231 | 16 | 89608702 | Yes | Yes | G | A | 0.369 | -0.01560 | 0.00264 | 3.44E-09 |
| rs4790874 | 17 | 1995177 | Yes | Yes | C | T | 0.532 | 0.01745 | 0.00255 | 8.43E-12 |
| rs11078713 | 17 | 7795972 | Yes | Yes | A | G | 0.419 | -0.01458 | 0.00258 | 1.59E-08 |
| rs28441558 | 17 | 7803118 | Yes | Yes | T | C | 0.056 | -0.03556 | 0.00553 | 1.24E-10 |
| rs11651955 | 17 | 16235462 | Yes | Yes | G | A | 0.499 | -0.01403 | 0.00255 | 3.74E-08 |
| rs67777803 | 17 | 27323322 | Yes | Yes | G | T | 0.172 | -0.02460 | 0.00338 | 3.18E-13 |
| rs2344976 | 17 | 30685935 | Yes | Yes | T | C | 0.612 | -0.01509 | 0.00261 | 7.98E-09 |
| rs3764351 | 17 | 37824339 | Yes | Yes | G | A | 0.657 | -0.01475 | 0.00268 | 3.89E-08 |
| rs72836318 | 17 | 44121579 | Yes | Yes | T | C | 0.246 | -0.01712 | 0.00296 | 7.00E-09 |
| rs17692129 | 17 | 44793283 | Yes | Yes | C | T | 0.331 | 0.01960 | 0.00271 | 4.57E-13 |
| rs75919030 | 17 | 50193197 | Yes | Yes | T | C | 0.267 | -0.02097 | 0.00288 | 3.35E-13 |
| rs2938134 | 17 | 50243397 | Yes | Yes | C | A | 0.673 | -0.01750 | 0.00278 | 3.14E-10 |
| rs2587507 | 17 | 77790135 | Yes | Yes | T | C | 0.502 | -0.01466 | 0.00255 | 8.69E-09 |
| rs34342129 | 18 | 5872472 | Yes | Yes | T | C | 0.509 | -0.01428 | 0.00255 | 2.13E-08 |
| rs4476253 | 18 | 25253297 | Yes | Yes | G | A | 0.240 | -0.01849 | 0.00298 | 5.78E-10 |
| rs7505855 | 18 | 31696075 | Yes | Yes | C | T | 0.586 | -0.01698 | 0.00259 | 5.31E-11 |
| rs8096225 | 18 | 36921851 | Yes | Yes | A | C | 0.703 | 0.01552 | 0.00279 | 2.63E-08 |
| rs67050670 | 18 | 39297254 | Yes | Yes | A | G | 0.229 | -0.02027 | 0.00303 | 2.34E-11 |
| rs2359180 | 18 | 41314171 | No | No | A | G | 0.369 | -0.01439 | 0.00264 | 4.98E-08 |
| rs72898831 | 18 | 42658643 | Yes | Yes | A | G | 0.155 | -0.02442 | 0.00352 | 4.14E-12 |
| rs8083764 | 18 | 49874515 | Yes | Yes | G | T | 0.306 | -0.01595 | 0.00276 | 7.97E-09 |
| rs1373178 | 18 | 49967811 | Yes | Yes | T | G | 0.588 | -0.02032 | 0.00259 | 4.16E-15 |
| rs62098013 | 18 | 50863861 | Yes | Yes | G | A | 0.365 | 0.01771 | 0.00265 | 2.24E-11 |
| rs72938304 | 18 | 53661743 | Yes | Yes | G | A | 0.113 | -0.02721 | 0.00402 | 1.36E-11 |
| rs11872397 | 18 | 72535282 | Yes | Yes | G | A | 0.253 | -0.01711 | 0.00293 | 5.20E-09 |
| rs71367544 | 18 | 77574374 | Yes | Yes | C | T | 0.203 | 0.02055 | 0.00317 | 8.54E-11 |
| rs76608582 | 19 | 4474725 | No | No | C | A | 0.049 | -0.03455 | 0.00591 | 4.88E-09 |
| rs10853981 | 19 | 4965064 | Yes | Yes | G | A | 0.330 | 0.01479 | 0.00271 | 4.88E-08 |
| rs113230003 | 19 | 18460956 | Yes | Yes | G | A | 0.255 | -0.01888 | 0.00292 | 1.05E-10 |
| rs8103660 | 19 | 18566395 | Yes | Yes | T | C | 0.354 | 0.01580 | 0.00266 | 3.03E-09 |
| rs117734003 | 19 | 51129745 | Yes | No | G | C | 0.067 | 0.03030 | 0.00509 | 2.57E-09 |
| rs1126757 | 19 | 55879872 | Yes | Yes | C | T | 0.473 | 0.01416 | 0.00255 | 2.92E-08 |
| rs6050446 | 20 | 25195509 | Yes | Yes | A | G | 0.971 | 0.05441 | 0.00761 | 8.80E-13 |
| rs6058782 | 20 | 29946968 | Yes | Yes | C | T | 0.908 | 0.02971 | 0.00442 | 1.78E-11 |
| rs1555445 | 20 | 31175258 | Yes | No | A | T | 0.318 | 0.01876 | 0.00274 | 7.75E-12 |
| rs6073075 | 20 | 42015801 | Yes | No | T | A | 0.824 | -0.01870 | 0.00335 | 2.44E-08 |
| rs910912 | 20 | 54462393 | Yes | Yes | T | C | 0.739 | -0.01677 | 0.00291 | 7.82E-09 |
| rs6011779 | 20 | 61984317 | Yes | Yes | C | T | 0.806 | -0.01918 | 0.00323 | 2.83E-09 |
| rs3810496 | 20 | 62406886 | Yes | Yes | T | C | 0.619 | 0.01588 | 0.00263 | 1.54E-09 |
| rs4818005 | 21 | 40588819 | Yes | Yes | G | A | 0.581 | -0.02043 | 0.00264 | 1.09E-14 |
| rs139896 | 22 | 38397797 | Yes | Yes | T | C | 0.648 | 0.01544 | 0.00267 | 7.14E-09 |
| rs4822102 | 22 | 42698430 | Yes | Yes | C | T | 0.618 | -0.01654 | 0.00262 | 2.78E-10 |
| rs9627272 | 22 | 46442288 | Yes | No | G | C | 0.407 | -0.01547 | 0.00259 | 2.42E-09 |
| **SNPs of smoking intensity** | | | | | | | | | | |
| RSID | Chromosome | Position | One-sample  MR | Two-sample  MR | Reference  allele | Effect  allele | Effect allele  frequency | Beta | Standard  error | *p*-value |
| rs11264100 | 1 | 35591626 | Yes | Yes | A | G | 0.876 | -0.02217 | 0.00370 | 2.22E-09 |
| rs2072659 | 1 | 154548521 | No | No | C | G | 0.099 | -0.02998 | 0.00410 | 2.51E-13 |
| rs34973462 | 1 | 175993820 | Yes | Yes | C | T | 0.334 | 0.01507 | 0.00259 | 5.85E-09 |
| rs7599488 | 2 | 60718347 | Yes | Yes | C | T | 0.437 | 0.01412 | 0.00245 | 8.95E-09 |
| rs78408772 | 2 | 62710608 | Yes | Yes | C | T | 0.102 | -0.02200 | 0.00402 | 4.51E-08 |
| rs10204824 | 2 | 148372720 | Yes | No | A | G | 0.639 | -0.01798 | 0.00253 | 1.35E-12 |
| rs2084533 | 3 | 16872929 | Yes | Yes | C | T | 0.321 | 0.01612 | 0.00261 | 6.53E-10 |
| rs7431710 | 3 | 48935583 | Yes | Yes | G | A | 0.654 | -0.01829 | 0.00257 | 1.04E-12 |
| rs2236951 | 3 | 50421081 | Yes | Yes | T | C | 0.200 | -0.01719 | 0.00304 | 1.59E-08 |
| rs699165 | 3 | 136224697 | Yes | Yes | A | G | 0.745 | 0.01612 | 0.00279 | 8.09E-09 |
| rs28813180 | 3 | 158083918 | Yes | Yes | G | A | 0.498 | -0.01550 | 0.00243 | 1.95E-10 |
| rs1024323 | 4 | 3006043 | Yes | Yes | C | T | 0.382 | -0.01442 | 0.00251 | 8.66E-09 |
| rs11940255 | 4 | 67086288 | Yes | Yes | G | A | 0.717 | -0.01716 | 0.00270 | 2.20E-10 |
| rs10454798 | 4 | 67980830 | Yes | Yes | G | T | 0.253 | 0.01584 | 0.00280 | 1.53E-08 |
| rs7766641 | 6 | 26184102 | Yes | Yes | G | A | 0.272 | -0.01728 | 0.00274 | 2.91E-10 |
| rs215600 | 7 | 32333642 | Yes | Yes | G | A | 0.645 | -0.02400 | 0.00254 | 4.02E-21 |
| rs62447179 | 7 | 50339609 | Yes | Yes | G | A | 0.298 | -0.01527 | 0.00266 | 9.68E-09 |
| rs2741351 | 8 | 27418040 | Yes | Yes | A | C | 0.826 | 0.01848 | 0.00321 | 8.80E-09 |
| rs73229090 | 8 | 27442127 | Yes | Yes | C | A | 0.112 | 0.02621 | 0.00386 | 1.14E-11 |
| rs13253502 | 8 | 42442018 | No | No | G | A | 0.407 | -0.01384 | 0.00248 | 2.31E-08 |
| rs4236926 | 8 | 42578059 | Yes | Yes | T | G | 0.766 | 0.03427 | 0.00288 | 7.66E-33 |
| rs790564 | 8 | 64604218 | Yes | Yes | A | C | 0.729 | -0.01762 | 0.00274 | 1.24E-10 |
| rs75596189 | 9 | 136468701 | Yes | Yes | C | T | 0.112 | 0.03580 | 0.00386 | 1.84E-20 |
| rs3025383 | 9 | 136502369 | Yes | Yes | T | C | 0.187 | -0.03138 | 0.00312 | 9.78E-24 |
| rs7951365 | 11 | 16377044 | Yes | Yes | T | C | 0.310 | 0.01776 | 0.00263 | 1.53E-11 |
| rs10742683 | 11 | 43667625 | Yes | Yes | G | A | 0.415 | -0.01349 | 0.00247 | 4.83E-08 |
| rs113001570 | 11 | 46737412 | Yes | No | A | T | 0.067 | 0.02980 | 0.00488 | 1.04E-09 |
| rs7125588 | 11 | 113436072 | Yes | Yes | A | G | 0.429 | -0.01690 | 0.00246 | 6.50E-12 |
| rs11846838 | 14 | 104184737 | Yes | No | G | A | 0.327 | 0.01518 | 0.00260 | 5.03E-09 |
| rs1115019 | 15 | 57141231 | Yes | Yes | T | C | 0.790 | -0.01786 | 0.00299 | 2.27E-09 |
| rs632811 | 15 | 59155050 | Yes | Yes | A | G | 0.330 | -0.01775 | 0.00278 | 1.67E-10 |
| rs4886550 | 15 | 78243579 | No | No | A | G | 0.288 | -0.01993 | 0.00340 | 4.58E-09 |
| rs12438181 | 15 | 78812098 | Yes | Yes | G | A | 0.218 | -0.01853 | 0.00298 | 4.97E-10 |
| rs10519203 | 15 | 78814046 | Yes | No | G | A | 0.655 | -0.09362 | 0.00259 | 4.97E-10 |
| rs28438420 | 15 | 78836288 | Yes | No | A | T | 0.554 | 0.01756 | 0.00247 | 1.25E-12 |
| rs72740955 | 15 | 78849779 | Yes | Yes | C | T | 0.337 | 0.03175 | 0.00260 | 2.42E-34 |
| rs146009840 | 15 | 78906177 | Yes | No | A | T | 0.335 | 0.02212 | 0.00261 | 2.00E-17 |
| rs28681284 | 15 | 78908565 | Yes | Yes | C | T | 0.210 | -0.04868 | 0.00302 | 2.10E-58 |
| rs8040868 | 15 | 78911181 | Yes | Yes | T | C | 0.400 | 0.01601 | 0.00251 | 1.79E-10 |
| rs3743063 | 15 | 79065171 | Yes | Yes | A | C | 0.562 | -0.01672 | 0.00248 | 1.53E-11 |
| rs182317 | 15 | 89943601 | Yes | Yes | G | T | 0.355 | -0.01559 | 0.00257 | 1.31E-09 |
| rs1592485 | 16 | 52093549 | Yes | Yes | C | A | 0.611 | -0.01615 | 0.00250 | 1.11E-10 |
| rs12924872 | 16 | 69552215 | Yes | Yes | C | T | 0.463 | -0.01341 | 0.00245 | 4.39E-08 |
| rs258321 | 16 | 89756473 | Yes | Yes | A | G | 0.429 | 0.01580 | 0.00247 | 1.53E-10 |
| rs4144686 | 18 | 53251725 | Yes | Yes | G | A | 0.167 | -0.01855 | 0.00326 | 1.35E-08 |
| rs4485470 | 18 | 62125063 | Yes | Yes | G | A | 0.592 | -0.01527 | 0.00248 | 7.05E-10 |
| rs59208569 | 19 | 4044424 | No | No | G | C | 0.829 | 0.02048 | 0.00323 | 2.45E-10 |
| rs143200968 | 19 | 41338847 | Yes | No | G | C | 0.025 | -0.08610 | 0.00786 | 6.97E-28 |
| rs56113850 | 19 | 41353107 | Yes | Yes | T | C | 0.555 | 0.05231 | 0.00247 | 4.01E-99 |
| rs8192726 | 19 | 41354496 | Yes | Yes | C | A | 0.068 | -0.03934 | 0.00489 | 8.35E-16 |
| rs117824460 | 19 | 41371480 | No | No | A | G | 0.026 | -0.09526 | 0.00773 | 7.66E-35 |
| rs6078373 | 20 | 11863500 | Yes | Yes | G | A | 0.402 | 0.01607 | 0.00248 | 9.40E-11 |
| rs1737894 | 20 | 31054702 | Yes | No | C | G | 0.408 | 0.01686 | 0.00248 | 9.90E-12 |
| rs2273500 | 20 | 61986949 | Yes | Yes | T | C | 0.147 | 0.03639 | 0.00344 | 3.49E-26 |
| rs7281463 | 21 | 40520783 | Yes | Yes | A | C | 0.413 | 0.01368 | 0.00247 | 3.15E-08 |
| **SNPs of age of smoking initiation** | | | | | | | | | | |
| RSID | Chromosome | Position | One-sample  MR | Two-sample  MR | Reference  allele | Effect  allele | Effect allele  frequency | Beta | Standard  error | *p*-value |
| rs7559982 | 2 | 63622309 | Yes | No | T | A | 0.564 | -0.01722 | 0.00244 | 1.67E-12 |
| rs72853300 | 2 | 145638766 | Yes | Yes | C | T | 0.153 | 0.01896 | 0.00336 | 1.75E-08 |
| rs12611472 | 2 | 225353649 | Yes | Yes | T | C | 0.297 | 0.01842 | 0.00265 | 3.48E-12 |
| rs11915747 | 3 | 85699040 | Yes | No | C | G | 0.354 | 0.02022 | 0.00254 | 1.57E-15 |
| rs624833 | 4 | 2881256 | Yes | Yes | T | G | 0.302 | 0.01575 | 0.00264 | 2.36E-09 |
| rs2471711 | 4 | 28589079 | Yes | Yes | C | T | 0.152 | -0.01922 | 0.00337 | 1.19E-08 |
| rs7682598 | 4 | 68000888 | Yes | Yes | A | G | 0.771 | 0.01726 | 0.00288 | 2.09E-09 |
| rs13136239 | 4 | 140908755 | Yes | Yes | G | A | 0.342 | 0.01481 | 0.00255 | 6.29E-09 |
| rs1403174 | 7 | 2032865 | Yes | No | A | T | 0.579 | 0.01550 | 0.00245 | 2.50E-10 |
| rs11780471 | 8 | 27344719 | Yes | No | G | A | 0.060 | 0.03296 | 0.00509 | 9.44E-11 |
| **SNPs of smoking cessation** | | | | | | | | | | |
| RSID | Chromosome | Position | One-sample  MR | Two-sample  MR | Reference  allele | Effect  allele | Effect allele  frequency | Beta | Standard  error | *p*-value |
| rs112187834 | 2 | 23953454 | Yes | No | T | A | 0.140 | 0.03341 | 0.00562 | 2.81E-09 |
| rs7617480 | 3 | 49210732 | Yes | Yes | A | C | 0.773 | -0.03287 | 0.00466 | 1.68E-12 |
| rs12203592 | 6 | 396321 | Yes | Yes | C | T | 0.176 | -0.02921 | 0.00512 | 1.21E-08 |
| rs707968 | 6 | 35058117 | Yes | Yes | A | G | 0.681 | 0.02327 | 0.00419 | 2.76E-08 |
| rs7778443 | 7 | 32314690 | Yes | Yes | T | C | 0.618 | -0.02300 | 0.00402 | 1.04E-08 |
| rs1565735 | 8 | 27426077 | Yes | No | T | A | 0.199 | -0.03456 | 0.00489 | 1.54E-12 |
| rs60749569 | 8 | 42602668 | Yes | No | A | T | 0.080 | -0.04007 | 0.00721 | 2.68E-08 |
| rs12378015 | 9 | 127917257 | Yes | Yes | G | A | 0.300 | -0.02766 | 0.00426 | 8.31E-11 |
| rs9409844 | 9 | 136461851 | Yes | Yes | G | A | 0.045 | -0.05856 | 0.00939 | 4.37E-10 |
| rs3025327 | 9 | 136467344 | Yes | No | G | C | 0.107 | 0.07859 | 0.00631 | 1.19E-35 |
| rs10821523 | 9 | 136473572 | Yes | No | A | C | 0.536 | 0.02616 | 0.00391 | 2.28E-11 |
| rs1611124 | 9 | 136509275 | Yes | Yes | G | T | 0.068 | -0.04534 | 0.00776 | 5.26E-09 |
| rs7109376 | 11 | 16372431 | Yes | No | T | A | 0.279 | 0.02806 | 0.00435 | 1.14E-10 |
| rs591143 | 15 | 47647755 | Yes | Yes | C | T | 0.592 | -0.02433 | 0.00399 | 1.14E-09 |
| rs3866543 | 15 | 76629609 | Yes | Yes | T | G | 0.523 | 0.02220 | 0.00391 | 1.35E-08 |
| rs518425 | 15 | 78883813 | Yes | Yes | A | G | 0.285 | -0.03050 | 0.00432 | 1.72E-12 |
| rs145580088 | 19 | 41342842 | Yes | Yes | A | G | 0.024 | 0.09096 | 0.01275 | 9.48E-13 |
| rs56113850 | 19 | 41353107 | Yes | Yes | T | C | 0.567 | -0.05761 | 0.00394 | 1.61E-48 |
| rs117824460 | 19 | 41371480 | No | No | A | G | 0.027 | 0.08650 | 0.01215 | 1.09E-12 |
| rs59586387 | 19 | 41375030 | Yes | No | C | G | 0.068 | 0.05139 | 0.00776 | 3.37E-11 |
| rs6011779 | 20 | 61984317 | Yes | Yes | C | T | 0.806 | -0.05002 | 0.00498 | 9.89E-24 |
| rs4809543 | 20 | 61986950 | Yes | Yes | G | A | 0.076 | 0.04441 | 0.00744 | 2.40E-09 |
| rs6089904 | 20 | 62018289 | Yes | No | A | T | 0.047 | -0.06418 | 0.00925 | 4.01E-12 |
| rs9607805 | 22 | 41854446 | Yes | Yes | C | T | 0.725 | 0.02954 | 0.00437 | 1.37E-11 |

**Supplemental Table 2.** Power analyses for binary traits

|  | Exposed  (cases/non-cases) | Non-exposed  (cases/non-cases) | Exposed participants  in non-cases | Alpha  risk | Beta  risk | Detectable  OR |
| --- | --- | --- | --- | --- | --- | --- |
| Smoking initiation,  women (*n* = 28,606) | 1,722/11,667 | 1,717/13,500 | 46.4% | 0.05 | 0.20 | ≥1.11 |
| Smoking initiation,  men (*n* = 27,096) | 1,688/11,518 | 1,607/12,303 | 48.4% | 0.05 | 0.20 | ≥1.11 |
| Smoking cessation,  women (*n* = 13,389) | 910/6,717 | 812/4,950 | 57.6% | 0.05 | 0.20 | ≤0.86 |

**Supplemental Table 3.** Power analyses for continuous traits

|  | Number of cases/non-cases | Alpha  risk | Beta  risk | Detectable  OR |
| --- | --- | --- | --- | --- |
| Smoking intensity,  women (*n* = 6,160) | 880/5,280 | 0.05 | 0.20 | ≥1.10 |
| Smoking intensity,  men (*n* = 5,307) | 753/4,554 | 0.05 | 0.20 | ≥1.11 |
| Age at smoking initiation, women  (*n* = 10,374) | 1,423/9,311 | 0.05 | 0.20 | ≤0.92 |

**Supplemental Table 4.** Comparison between participants with and without genotype information

|  | **Women** | | | **Men** | | |
| --- | --- | --- | --- | --- | --- | --- |
|  | Included | Non-included | *p*-value | Included | Non-included | *p*-value |
| Age (years), mean ± standard deviation | 30.3 ± 4.16 | 30.1 ± 4.76 | <0.001 | 32.7 ± 4.91 | 32.7 ± 5.61 | 0.690 |
| Education years,  mean ± standard deviation | 17.5 ± 3.12 | 16.9 ± 3.44 | <0.001 | 16.6 ± 3.50 | 15.9 ± 3.65 | <0.001 |
| Body mass index (kg/m^2^),  median (25^th^-75^th^ percentile) | 23.1  (21.2-25.9) | 23.1  (21.1-26.0) | 0.125 | 25.5  (23.7-27.7) | 25.4  (23.6-27.7) | <0.001 |
| Previous pregnancies,  *n* (%): |  |  | 0.379 |  |  | 0.045 |
| 0 | 13,016 (45.0%) | 31,718 (44.7%) |  | 12,408 (45.3%) | 32,326 (44.6%) |  |
| 1 or more | 15,902 (55.0%) | 39,236 (55.3%) |  | 14,979 (54.7%) | 40,159 (55.4%) |  |
| Ever smokers (all participants),  *n* (%): | 13,575 (46.9%) | 36,642 (52.5%) | <0.001 | 13,353 (48.7%) | 34,013 (46.9%) | <0.001 |
| Cigarettes/week (current smokers), median (25^th^-75^th^ percentile) | 35.0  (7.00-70.0) | 49.0  (10.0-91.0) | <0.001 | 56.0  (7.00-105) | 70.0  (10.0-105) | <0.001 |

**Supplemental Table 5.** Stratified association between smoking-related traits and infertility subtypes

|  | **All infertility cases** | | | **Infertility, non-ART users** | | **Infertility, ART users** | |
| --- | --- | --- | --- | --- | --- | --- | --- |
|  | **Fertile**  **(*n*)** | **Infertile**  **(*n*)** | **OR**  **(95% CI)** | **Proportion**  **(*n*, %)** | **OR**  **(95% CI)** | **Proportion**  **(*n*, %)** | **OR**  **(95% CI)** |
| **Women** | | | | | | | |
| Having ever smoked  (vs. never having smoked) | 25,167 | 3,439 | 1.03  (0.95 to 1.11) | 2,729  (79.4%) | 1.07  (0.98 to 1.16) | 710  (20.6%) | 0.89  (0.75 to 1.05) |
| +1 SD in the number of cigarettes smoked per week (current smokers) | 5,280 | 880 | 1.12  (1.03 to 1.21) | 760  (86.4%) | 1.12  (1.04 to 1.22) | 120  (13.6%) | 1.05  (0.86 to 1.29) |
| +1 SD in the age of smoking initiation (current + former smokers) | 9,311 | 1,423 | 0.89  (0.84 to 0.95) | 1,157  (81.3%) | 0.89  (0.83 to 0.95) | 266  (18.7%) | 0.92  (0.80 to 1.06) |
| Quitting smoking (vs. no cessation) (current + former smokers) | 11,667 | 1,722 | 0.83  (0.75 to 0.93) | 1,395  (81.0%) | 0.71  (0.63 to 0.80) | 327  (19.0%) | 1.82  (1.40 to 2.36) |
| **Men** | | | | | | | |
| Having ever smoked  (vs. never having smoked) | 23,821 | 3,275 | 1.04  (0.96 to 1.12) | 2,590  (79.1%) | 1.06  (0.97 to 1.15) | 685  (20.9%) | 0.96  (0.81 to 1.14) |
| +1 SD in the number of cigarettes smoked per week (current smokers) | 4,554 | 753 | 1.08  (0.99 to 1.18) | 629  (83.5%) | 1.06  (0.97 to 1.16) | 124  (16.5%) | 1.15  (0.96 to 1.37) |

**Supplemental Table 6**. Observational analyses of the associations between smoking-related traits and the odds of infertility adding further covariates in women.

| **Model definition** | Having ever smoked  (vs. never having smoked) (all participants,  *n* = 28,606)  OR [95% CI] | +1 SD in the number of cigarettes smoked per week (current smokers,  *n* = 6,160)  OR [95% CI] | +1 SD in the age of smoking initiation (current + former smokers, *n* = 10,734)  OR [95% CI] | Quitting smoking  (vs. no cessation) (current + former smokers, *n* = 13,389)  OR [95% CI] |
| --- | --- | --- | --- | --- |
| Model 1: non-adjusted | 1.16 (1.08 to 1.25) | 1.19 (1.11 to 1.28) | 0.94 (0.88 to 0.99) | 0.83 (0.75 to 0.91) |
| Model 2: adjusted for age,  years of education, BMI, and  number of previous pregnancies | 1.03 (0.95 to 1.11) | 1.12 (1.03 to 1.21) | 0.89 (0.84 to 0.95) | 0.83 (0.75 to 0.93) |
| Model 3: model 2 + smoking  trait in the father | 1.00 (0.92 to 1.09) | 1.10 (1.01 to 1.19) | - | - |
| Model 4: model 2 + diet quality  (fiber intake) | 1.03 (0.95 to 1.12) | 1.13 (1.04 to 1.22) | 0.90 (0.84 to 0.96) | 0.82 (0.74 to 0.92) |
| Model 5: model 2 + physical  activity | 1.03 (0.95 to 1.12) | 1.12 (1.04 to 1.21) | 0.89 (0.83 to 0.95) | 0.83 (0.75 to 0.93) |
| Model 6: model 2 + caffeine intake | 1.01 (0.93 to 1.10) | 1.08 (1.00 to 1.17) | 0.90 (0.84 to 0.96) | 0.85 (0.76 to 0.95) |
| Model 7: model 2 + alcohol use | 1.08 (0.99 to 1.17) | 1.11 (1.02 to 1.20) | 0.89 (0.84 to 0.95) | 0.81 (0.72 to 0.90) |
| Model 8: model 2 + occupational features | 1.03 (0.95 to 1.11) | 1.12 (1.04 to 1.22) | 0.88 (0.83 to 0.94) | 0.83 (0.74 to 0.92) |
| Model 9: model 2 + diet quality,  physical activity, caffeine intake, alcohol use, and occupational features | 1.05 (0.96 to 1.14) | 1.10 (1.01 to 1.21) | 0.90 (0.84 to 0.96) | 0.81 (0.72 to 0.91) |
| Model 10: model 2 + diet quality,  physical activity, caffeine intake, alcohol use, and occupational features, and smoking trait in the father | 1.03 (0.94 to 1.13) | 1.08 (0.99 to 1.19) | - | - |

**Supplemental Table 7**. Observational analyses of the associations between smoking-related traits and the odds of infertility adding further covariates in men

| **Model definition** | Having ever smoked  (vs. never having smoked)  (all participants,  *n* = 27,096)  OR [95% CI] | +1 SD in the number of cigarettes smoked per week (current smokers, *n* = 5,307)  OR [95% CI] |
| --- | --- | --- |
| Model 1: non-adjusted | 1.11 (1.03 to 1.20) | 1.12 (1.04 to 1.21) |
| Model 2: adjusted for age,  years of education, BMI, and  number of previous pregnancies | 1.04 (0.96 to 1.12) | 1.08 (0.99 to 1.18) |
| Model 3: model 2 + smoking  trait in the mother | 1.02 (0.94 to 1.11) | 1.05 (0.96 to 1.15) |
| Model 4: model 2 + diet quality  (fiber intake) | 1.03 (0.91 to 1.16) | 1.15 (1.00 to 1.32) |
| Model 5: model 2 + physical  activity | 1.03 (0.95 to 1.12) | 1.08 (0.99 to 1.18) |
| Model 6: model 2 + caffeine  intake | 1.02 (0.90 to 1.15) | 1.15 (1.00 to 1.32) |
| Model 7: model 2 + alcohol  consumption | 1.04 (0.96 to 1.13) | 1.08 (0.99 to 1.17) |
| Model 8: model 2 + occupational features | 1.03 (0.95 to 1.12) | 1.08 (0.99 to 1.18) |
| Model 9: model 2 + diet quality,  physical activity, caffeine intake, alcohol use,  and occupational features | 1.01 (0.89 to 1.14) | 1.15 (1.00 to 1.32) |
| Model 10: model 2 + diet quality,  physical activity, caffeine intake, alcohol use,  and occupational features,  and smoking trait in the mother | 1.01 (0.88 to 1.15) | 1.13 (0.97 to 1.30) |

**Supplemental Table 8.** Linear associations between genetic risk scores and infertility risk factors.

|  | Age (years) | Education (years) | Body mass index (kg/m^2^) | Previous pregnancies (n) |  |
| --- | --- | --- | --- | --- | --- |
| **Women** | | | | | |
| Smoking initiation GRS  (Δ1 SD) | -0.13 (-0.18 to -0.072)  (*p* < 0.001) | -0.22 (-0.26 to -0.17)  (*p* < 0.001) | 0.16 (0.10 to 0.22)  (*p* < 0.001) | 0.008 (-0.001 to 0.017)  (*p* = 0.087) |  |
| Smoking intensity GRS  (Δ1 SD) | -0.067 (-0.18 to 0.048)  (*p* = 0.253) | -0.026 (-0.12 to 0.069)  (*p* = 0.589) | -0.056 (-0.17 to 0.059)  (*p* = 0.338) | 0.003 (-0.017 to 0.023)  (*p* = 0.769) |  |
| Age of smoking initiation GRS  (Δ1 SD) | 0.082 (-0.008 to 0.17)  (*p* = 0.074) | 0.082 (0.009 to 0.15)  (*p* = 0.028) | -0.068 (-0.16 to 0.027)  (*p* = 0.162) | 0.003 (-0.012 to 0.017)  (*p* = 0.726) |  |
| Smoking cessation GRS  (Δ1 SD) | 0.029 (-0.052 to 0.11)  (*p* = 0.485) | 0.078 (0.014 to 0.14)  (*p* = 0.017) | 0.024 (-0.057 to 0.10)  (*p* = 0.565) | 0.010 (-0.012 to 0.014)  (*p* = 0.875) |  |
| **Men** | | | | | |
| Smoking initiation GRS  (Δ1 SD) | -0.006 (-0.072 to 0.060)  (*p* = 0.866) | -0.25 (-0.29 to -0.20)  (*p* < 0.001) | 0.20 (0.16 to 0.25)  (*p* < 0.001) | -0.007 (-0.016 to 0.003)  (*p* = 0.152) |  |
| Smoking intensity GRS  (Δ1 SD) | 0.13 (-0.026 to 0.28)  (*p* = 0.103) | -0.091 (-0.20 to 0.014)  (*p* = 0.090) | -0.073 (-0.18 to 0.030)  (*p* = 0.163) | -0.004 (-0.024 to 0.017)  (*p* = 0.712) |  |

**Supplemental Table 9.** Multivariable and age-stratified one-sample MR analyses

|  | One-sample MR  (main analyses) | Multivariable MR (accounting for education years and body mass index) | MR: age of delivery  < median | MR: age of delivery  > median |
| --- | --- | --- | --- | --- |
| **Women** | | | | |
| Δ1 SD in the genetically predicted likelihood of smoking initiation  (all participants) | 1.01  (0.98 to 1.05) | 1.00  (0.94 to 1.05) | 1.06  (1.00 to 1.12) | 0.99  (0.95 to 1.04) |
| Δ1 SD in the genetically predicted number of cigarettes smoked/week (current smokers) | 0.96  (0.89 to 1.03) | 0.97  (0.90 to 1.05) | 0.97  (0.87 to 1.07) | 0.96  (0.86 to 1.06) |
| Δ1 SD in the genetically predicted age of smoking initiation  (current + former smokers) | 0.96  (0.91 to 1.02) | 0.99  (0.93 to 1.04) | 0.96  (0.89 to 1.05) | 0.96  (0.89 to 1.04) |
| Δ1 SD in the genetically predicted likelihood of smoking cessation  (current + former smokers) | 1.04  (0.99 to 1.09) | 1.11  (0.96 to 1.27) | 1.03  (0.96 to 1.11) | 1.04  (0.97 to 1.12) |
| **Men** | | | | |
| Δ1 SD in the genetically predicted likelihood of smoking initiation  (all participants) | 0.99  (0.95 to 1.03) | 0.95  (0.90 to 1.01) | 1.00  (0.94 to 1.06) | 0.98  (0.94 to 1.03) |
| Δ1 SD in the genetically predicted number of cigarettes smoked/week (current smokers) | 1.02  (0.95 to 1.10) | 0.94  (0.87 to 1.02) | 1.02  (0.91 to 1.14) | 1.01  (0.91 to 1.12) |

**Supplemental Table 10.** Associations between 1 SD increases in GRSs for smoking traits and infertility in non-exposed participants (no-relevance sensitivity analyses)

| **Women** | |
| --- | --- |
| Δ1 SD in the genetically predicted  number of cigarettes smoked/week  (never + former smokers) | 0.96  (0.92 to 1.01) |
| Δ1 SD in the genetically predicted  age of smoking initiation  (never smokers) | 1.01  (0.96 to 1.06) |
| Δ1 SD in the genetically predicted  likelihood of smoking cessation  (never smokers) | 0.98  (0.93 to 1.04) |
| **Men** | |
| Δ1 SD in the genetically predicted  number of cigarettes smoked/week  (never + former smokers) | 0.99  (0.95 to 1.04) |

**Supplemental Table 11.** Estimates (odds ratios) of all two-sample Mendelian randomization methods plus indicators of horizontal pleiotropy and SNP heterogeneity.

|  | Inverse  variance  weighted | MR Egger | Weighted  median | Weighted  mode | MR-Robust Adjusted  Profile Score | Horizontal  pleiotropy  (MR Egger) | Cochran’s  Q | Rücker’s  Q’ |
| --- | --- | --- | --- | --- | --- | --- | --- | --- |
| **Women** | | | | | | | | |
| Δ1 SD in the genetically predicted likelihood of smoking initiation | 0.90  (0.75 to 1.09) | 1.15  (0.52 to 2.54) | 0.98  (0.74 to 1.30) | 1.12  (0.50 to 2.50) | 0.99  (0.83 to 1.19) | *p* = 0.536 | 249  (*p* = 0.985) | 249  (*p* = 0.984) |
| Δ1 SD in the genetically predicted number of cigarettes smoked/week | 0.67  (0.39 to 1.14) | 0.37  (0.098 to 1.39) | 0.64  (0.30 to 1.35) | 0.72  (0.30 to 1.74) | 0.66  (0.44 to 1.01) | *p* = 0.343 | 62.5  (*p* = 0.022) | 61.1  (*p* = 0.023) |
| Δ1 SD in the genetically predicted age of smoking initiation | 0.46  (0.11 to 1.99) | 1.81  (8×10^-4^ to 4,100) | 0.27  (0.049 to 1.50) | 0.23  (0.024 to 2.11) | 0.65  (0.20 to 2.09) | *p* = 0.737 | 7.42  (*p* = 0.284) | 7.24  (*p* = 0.204) |
| Δ1 SD in the genetically predicted likelihood of smoking cessation | 0.74  (0.41 to 1.33) | 0.65  (0.12 to 3.63) | 1.21  (0.59 to 2.49) | 1.47  (0.52 to 4.13) | 0.81  (0.53 to 1.23) | *p* = 0.870 | 19.4  (*p* = 0.196) | 19.4  (*p* = 0.152) |
| **Men** | | | | | | | | |
| Δ1 SD in the genetically predicted likelihood of smoking initiation | 0.92  (0.76 to 1.11) | 0.87  (0.38 to 2.01) | 0.94  (0.70 to 1.26) | 0.77  (0.32 to 1.84) | 0.91  (0.76 to 1.09) | *p* = 0.907 | 230  (*p* = 0.999) | 230  (*p* = 0.999) |
| Δ1 SD in the genetically predicted number of cigarettes smoked/week | 1.10  (0.78 to 1.56) | 0.77  (0.42 to 1.43) | 0.88  (0.52 to 1.51) | 0.94  (0.58 to 1.54) | 1.03  (0.73 to 1.44) | *p* = 0.179 | 35.8  (*p* = 0.775) | 33.9  (*p* = 0.809) |

**Supplemental Table 12.** Instruments for the estimation of fiber intake.

| **Food** | **Average**  **serving weight** | **Fiber quantity**  **per 100g of food** |
| --- | --- | --- |
| White bread | 45 g (per slice) | 2 g |
| Medium coarse-grain bread | 45 g (per slice) | 4.5 g |
| Full coarse-grain bread | 45 g (per slice) | 6 g |
| Crispbread | 15 g (per slice) | 14 g |
| Jam or jelly | 20 g | 2 g |
| Italian salad (spread) | 20 g | 1 g |
| Vegetarian dishes | 180 g | 3 g |
| Sausage/burgers | 180 g | 1 g |
| Pizza | 300 g | 2.5 g |
| Kebab | 75 g of salad  per serving | 1.6 g^1^ |
| Salad | 150 g | 1.6 g^1^ |
| Cooked vegetables | 175 g | 2 g |
| Vegetable soup | 75 g of cooked  vegetables per serving | 2 g |
| Fruit | 150 g | 2.5 g^2^ |

1. We assumed the following food proportions in a standard salad: 50% lettuce/green leafy vegetables (1 g of fiber/100 g), 10% cabbage (3 g of fiber/100 g), 10% carrot (2 g of fiber/100 g), 10% onion (2 g of fiber/100 g), 10% pepper (2 g of fiber/100 g) and 10% tomato (2 g of fiber/100 g).

2. Average fiber content in fruits usually consumed in Norway (apples, apricots, banana, blackberries, blackcurrants, blueberries, cherries, clementine, cranberries, grapefruit, grapes, kiwifruit, kumquat, lemon, lime, mango, melon, nectarine, orange, peach, pear, persimmon, pineapple, plum, pomegranate, pomelo, raspberries, redcurrants, and strawberries).

**Supplemental Table 13.** Instruments for the estimation of the energy expenditure in physical activity.

| **Type of physical activity** | **Average duration**  **of a session** | **Energy**  **expenditure** |
| --- | --- | --- |
| Walking | 60 minutes | 3 METs-min |
| Brisk walking | 45 minutes | 4.5 METs-min |
| Running or jogging | 45 minutes | 9 METs-min |
| Cycling | 90 minutes | 9 METs-min |
| Weight training | 45 minutes | 8 METs-min |
| Aerobics for pregnant women | 45 minutes | 5 METs-min |
| Mid-intensity aerobics | 45 minutes | 6 METs-min |
| High-intensity aerobics | 45 minutes | 7 METs-min |
| Dancing (rock, swing) | 45 minutes | 5.5 METs-min |
| Cross-country skiing | 120 minutes | 9 METs-min |
| Ball sports (reference: basketball) | 45 minutes | 8 METs-min |
| Swimming | 45 minutes | 8 METs-min |
| Horse riding | 45 minutes | 5 METs-min |

**SUPPLEMENTAL FIGURES**

**Supplemental Figure 1.** Two-sample Mendelian randomization scatterplots for the associations of smoking initiation (A), age of smoking initiation (B), smoking cessation (C), and smoking intensity in women (D), and smoking initiation (E) and smoking intensity in men (F) with infertility.


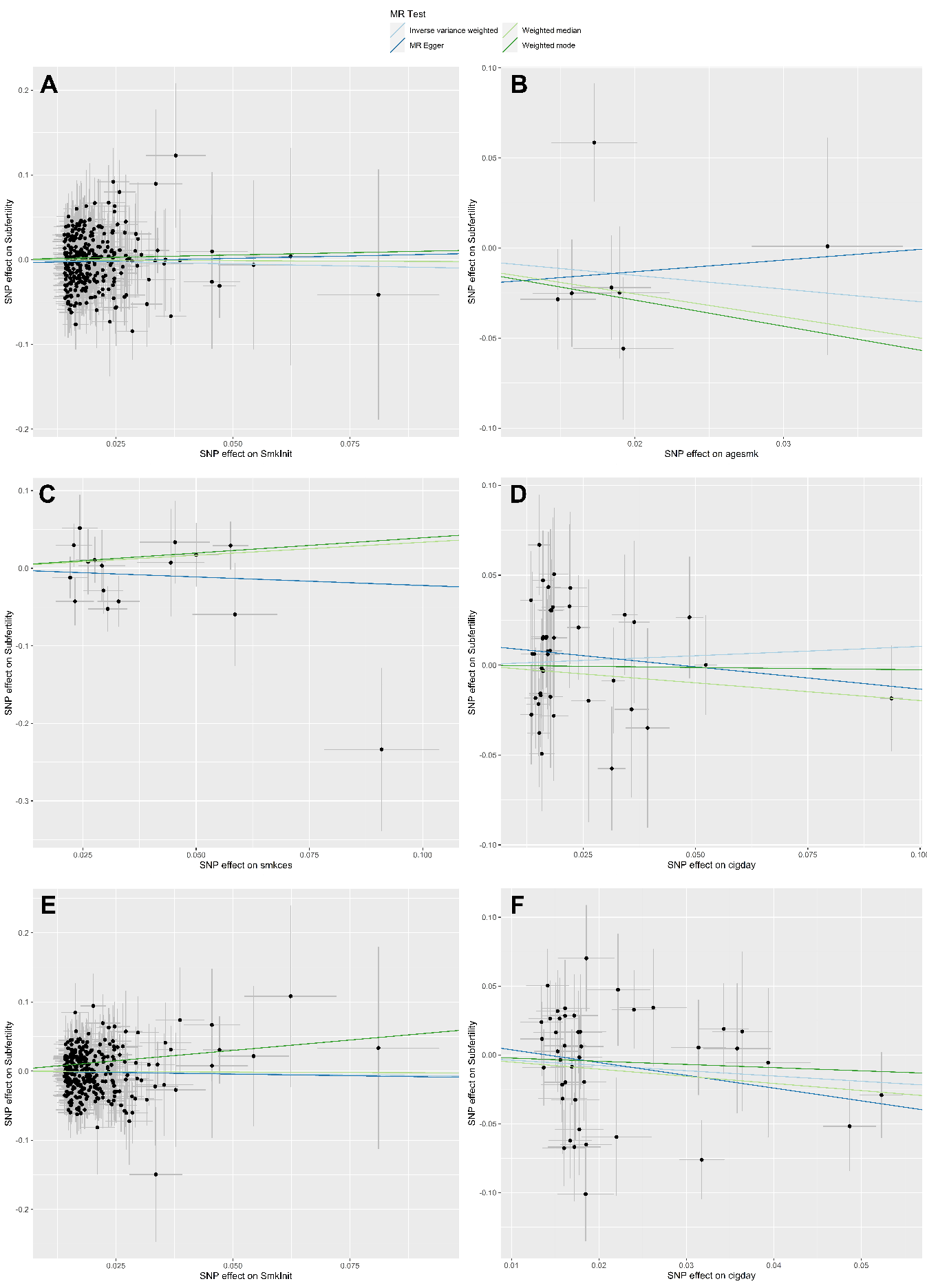


**SUPPLEMENTAL REFERENCES**

1. Englund-Ögge L, Birgisdottir BE, Sengpiel V, Brantsæter AL, Haugen M, Myhre R *et al.* Meal frequency patterns and glycemic properties of maternal diet in relation to preterm delivery: Results from a large prospective cohort study. PLoS One 2017;12:e0172896.

2. Qiu C, Coughlin KB, Frederick IO, Sorensen TK, Williams MA. Dietary fiber intake in early pregnancy and risk of subsequent preeclampsia. Am J Hypertens 2008;21:903-9.

3. Lund-Blix NA, Tapia G, Mårild K, Brantsæter AL, Eggesbø M, Mandal S *et al.* Maternal fibre and gluten intake during pregnancy and risk of childhood celiac disease: the MoBa study. Sci Rep 2020;10:16439.

4. Norwegian Department of Health. Weights, measures and portion sizes for foods. 2015.

5. Norwegian Department of Health. The Norwegian Food Composition Table (Matvaretabellen). 2021.

6. Ainsworth BE, Haskell WL, Leon AS, Jacobs DR, Jr., Montoye HJ, Sallis JF *et al.* Compendium of physical activities: classification of energy costs of human physical activities. Med Sci Sports Exerc 1993;25:71-80.

7. Sengpiel V, Elind E, Bacelis J, Nilsson S, Grove J, Myhre R *et al.* Maternal caffeine intake during pregnancy is associated with birth weight but not with gestational length: results from a large prospective observational cohort study. BMC Med 2013;11:42.

8. Zuccolo L, DeRoo LA, Wills AK, Davey Smith G, Suren P, Roth C *et al.* Pre-conception and prenatal alcohol exposure from mothers and fathers drinking and head circumference: results from the Norwegian Mother-Child Study (MoBa). Sci Rep 2016;7:39535.

9. Helgeland Ø, Vaudel M, Juliusson PB, Lingaas Holmen O, Juodakis J, Bacelis J *et al.* Genome-wide association study reveals dynamic role of genetic variation in infant and early childhood growth. Nat Commun 2019;10:4448.

10. Sole-Navais P, Bacelis J, Helgeland Ø, Modzelewska D, Vaudel M, Flatley C *et al.* Autozygosity mapping and time-to-spontaneous delivery in Norwegian parent-offspring trios. Hum Mol Genet 2021;29:3845-58.

11. Chang CC, Chow CC, Tellier LC, Vattikuti S, Purcell SM, Lee JJ. Second-generation PLINK: rising to the challenge of larger and richer datasets. Gigascience 2015;4:7.

12. Mägi R, Morris AP. GWAMA: software for genome-wide association meta-analysis. BMC Bioinformatics 2010;11:288.
